# Advancing Neuropsychological Rehabilitation in Primary Progressive Aphasia Based on Principles of Cognitive Neuroscience: A Scoping Review and Systematic Analysis of the Data

**DOI:** 10.3390/brainsci14121234

**Published:** 2024-12-08

**Authors:** Evgenia Gkintoni, Emilia Michou

**Affiliations:** 1 Department of Psychiatry, University General Hospital of Patras, 26504 Patras, Greece; 2 Department of Speech and Language Therapy, University of Patras, 26504 Patras, Greece; emiliamichou@upatras.gr

**Keywords:** primary progressive aphasia (PPA), neuropsychological rehabilitation, cognitive neuroscience, neuroplasticity, language improvement, language abilities, cognitive training, EEG, fMRI, neuroimaging

## Abstract

**Background/Objectives:** This systematic review of neuropsychological rehabilitation strategies for primary progressive aphasia will consider recent developments in cognitive neuroscience, especially neuroimaging techniques such as EEG and fMRI, to outline how these tools might be integrated into clinical practice to maximize treatment outcomes. **Methods:** A systematic search of peer-reviewed literature from the last decade was performed following the PRISMA guidelines across multiple databases. A total of 63 studies were included, guided by predefined inclusion and exclusion criteria, with a focus on cognitive and language rehabilitation in PPA, interventions guided by neuroimaging, and mechanisms of neuroplasticity. **Results:** Integration of neuroimaging techniques contributes to the increase in the efficacy of interventions with critical information about the neural mechanisms underlying language deficits in the aphasias. Traditional rehabilitation strategies, technology-assisted interventions, and non-invasive brain stimulation techniques hold considerable promise for language improvement. Neuroimaging was also found to be necessary in subtype-specific differentiation toward tailoring therapeutic intervention. Evidence also shows that directed and sustained interventions using neuroplasticity can have long-term effects in managing the symptoms of PPA. **Conclusions:** The present review underlines the necessity of including cognitive neuroscience techniques within neuropsychological rehabilitation to enhance therapeutic outcomes in PPA. In addition, neuroimaging modalities such as EEG and fMRI are also of great importance in understanding the underlying neurobiology of language disturbances and guiding tailored interventions. Long-term benefits of these approaches should be evaluated, including their applicability in routine clinical practice.

## 1. Introduction

Primary progressive aphasia (PPA) is an acquired neurodegenerative syndrome that leads to language disturbance and impacts quality of life. PPA decreases the life quality of patients since it causes impairments in social communication goals and ruptures important relations. Rehabilitation in people diagnosed with PPAS has been evolving in the latest years, providing insights for the appropriate methods as well as brain plasticity measures [1,2,3]. There are only a few intervention studies focused on PPA, and far less is known about the rehabilitation of scene descriptions in PPA than about rehabilitation of single-word processing. Research on neuropsychological rehabilitation for patients with PPA to promote communication has made some progress in recent years, but many questions remain, and developing interventions is often an iterative and exploratory process [4,5,6,7,8].

Primary progressive aphasia (PPA) refers to a syndromic entity whose clinical features are primary language deficits, except for agrammatism, presenting in a progressive and insidious manner. The 2011 classification established three variants for PPA, as follows: the non-fluent agrammatic variant (nfvPPA) defined by speech apraxia and/or agrammatism, the semantic variant (svPPA) delineated by the loss of word meanings and surface dyslexia, and the logopenic variant (lvPPA) characterized by speech hesitations, impaired repetition, and phonological awareness deficits. nfvPPA, svPPA, or lvPPA are also linked to different focal cortical atrophy and underlying proteinopathies, with nfvPPA correlated with the 4-repeat (4R) tau pathology, lvPPA associated with Alzheimer’s disease pathology, and svPPA related to the TDP-43 type C pathology [9,10]. Although variable labeling and research frameworks across history have been introduced, the nfvPPA/subcortical description is commonly identified as a progressive right hemisphere syndrome, with some subtle semantic impairment, especially in the domains of metaphor comprehension and complex sentence interpretation. Concerning its prevalence, cumulative global nfvPPA prevalence in prospective and retrospective multidomain dementias cohorts stands around 16%, representing almost a third of a miscellanea of presentations with impaired language and social cognition [11,12,13].

Individuals with primary progressive aphasia (PPA) display varying profiles of language and associated cognitive impairments related to different neurodegenerative diseases. The advent of therapies to treat aphasia associated with stroke has driven a more focused search for evidence-based interventions in PPA. However, evidence-based guidelines for neuropsychological interventions in PPA are not yet available, but they have the potential to impact the quality of life for a group of patients who are relatively young and previously unable to be helped by available treatments. In many cases, neuropsychological rehabilitation has been challenging and less effective because of the heterogeneity of the PPA syndrome and its clinical manifestations. Thus, the establishment of a consensus across different institutions and the promotion of training in this specific field can provide these undeniably needed standards in terms of practice and academic research [14].

PPA is a subset of progressive aphasias, rather than a variant of frontotemporal dementia, that is characterized by an insidious development of progressive decline affecting language-related activities of daily living over an interval of at least two years in the absence of other neurological or psychiatric illnesses, preceding the onset of memory, behavior, and visual impairments by at least two years. In the South African context, PPA is most seen in the older patient group and is often accompanied by additional signs of language deficits. Individuals with PPA have neurodegeneration, which primarily involves the language-dominant left half of the brain and is often right-handed. This supports the fact that language impairment is likely to considerably influence cognitive performance in these patients. To date, only two studies have considered the potential for rehabilitation in PPA, in the form of two case studies with conflicting results [15,16].

Primary progressive aphasia (PPA) is a disorder that primarily affects language, speech, and communication because of focal neurodegeneration. Neuropsychological deficits of PPA include language and communication impairments that affect listening, reading, speaking, and writing, cognitive impairments such as apraxia, visuospatial deficits, or executive control, and emotional recognition and production. The disorders in people with PPA lead to several potential social, psychological, and quality of life issues. Primary progressive aphasia (PPA) is a language-dominant, focal neurodegenerative disorder with a prevalence of 6 out of 100,000. Patients with PPA typically start the disease in their 50s/60s and typically survive another 8 years. The literature differentiates three clinical variants of PPA in accordance with the guidelines for the classification of PPA developed by a group of international experts. Regardless of the etiological heterogeneity, PPA has some consistent features, including decline in cognitive functions other than language and pervasive management of treatment and rehabilitation required to improve the quality of life of patients and caregivers. Therefore, the present study was performed to analyze the progress in the field of neuropsychological rehabilitation of individuals with PPA [1,17].

Primary progressive aphasia (PPA) is a devastating neurodegenerative syndrome that affects mostly younger individuals. The condition is slowly progressive and results in a variety of language and communication deficits. There are three broad classes of the condition: non-semantic PPA, semantic PPA, and logopenic PPA. Each of the PPA variants has somewhat unique features. Although people with non-fluent PPA are often hesitant, they can still produce powerful and effective communicative interactions. On the other hand, compared with people with non-fluent PPA, individuals with a severe form of the condition or with agrammatism or apraxia of speech in isolation become mostly silent. They show little voluntary communication and a striking paucity of speech (that is often termed “selective mutism”) [5,7].

Impairments in any or all of these associated domains can have marked consequences for the individual’s capacity for independence, social engagement, and well-being. Importantly, PPA results from progressive damage to neural networks that stretch well beyond “language areas”. This complexity in neuropsychological profile is an important contributory factor to “rehabilitation failure” when an exclusively neuropsychological approach is taken. Moreover, spared cognitive-linguistic abilities are necessary for a patient response to a rehabilitation program focused on those spared abilities. To address these complex cognitive-linguistic issues, a shift from insights informed by the field of neuropsychology to insights from cognitive neuroscience has occurred in the field of neurorehabilitation in the last five years. Neuropsychological rehabilitation incorporating insights from cognitive neuroscience is the field of neuropsychological rehabilitation that emphasizes patient-tailored treatments based on current knowledge on the normal, adaptive cognitive neuroscience at the root of an experimental rehabilitation intervention [11,18].

Cognitive impairments associated with PPA may affect individuals at different linguistic stages, which are not exclusive to brain areas but are synthetically presented by the current taxonomy. Impairments at the word and sentence level are likely to compromise the phonological, semantic, and morphosyntactic subcomponents due to the spread of degeneration in the connected networks that support single word and sentence processing. At the discourse level, PPA patients show word-finding difficulties, impeding topic development and integration, beyond a reduction in lexical-semantic fluency paralleled by a difficulty in manipulatable noun–attribute combinations and associative relationships. Non-linguistic performance may be normal, or those with logopenic PPA may show deficits in visual short-term memory and visuospatial function. Language comprehension of syntactically complex sentences is often impaired, even in agrammatic PPA types, unlike healthy elderly subjects showing syntactic deficits only when tasks become more demanding. Syntactic comprehension difficulty is mainly related to connected speech comprehension, but it can also appear when linguistic material is presented per modality (i.e., as a single linguistic input), as syntactic comprehension is intertwined with syntactic decoding processes [19,20,21].

Further impairments other than language are crucial for their subtle nature and role in shaping semantic access and word knowledge. Executive dysfunctions are reported in “phonological PPA” patients, a descriptive term used in this study to denote cases with prominent phonological processing deficits. These align with characteristics of the logopenic variant of PPA (lvPPA) but are emphasized here to highlight specific rehabilitation targets for phonological impairments. Patients with early executive deficits for verbal fluency tasks and an impetus for constraining semantic-access-driven behavior often show these features. Behavioral disturbances do occur and are potentially impairing. Apathy, depression, anxiety, and irritability occur at a similar frequency across the three PPA variants. Behavioral disturbances can consist of impulsiveness, disinhibition, and compulsivity, on the grounds of PPA patients being master processors. PPA patients have poor insight, and the decreased self-awareness is associated with specific areas of brain atrophy [21,22,23].

A handful of studies have suggested that speech and language therapy may confer potential benefits for some individuals with PPA, particularly those with the logopenic and nonfluent agrammatic syndromes. Traditional approaches to rehabilitation in PPA have taken a neurolinguistic framework, focusing on the use of semantic, phonological, pragmatic, and other language compensation techniques. A systematic analysis performed in 2023 [24] demonstrated promising results using traditional behavioral interventions (i.e., therapy, self-training) aimed at improving object naming performance. In comparison to the Randomized Control Trials (RCTs), the effects of traditional PPA rehabilitation are generally very mild (small effect sizes based on current meta-analysis) and the sustainability of these interventions is, however, very poorly investigated [24,25,26,27,28].

Within recent years, emerging technology-assisted interventions have recently shown preliminary evidence of feasibility, safety, and efficacy. New brain rehabilitation techniques for PPA include the use of repetitive transcranial magnetic stimulation (rTMS) and transcranial direct current stimulation (tDCS) to modulate the excitability of underlying cortical areas. Improvements on object naming following stimulation have extended up to 6 months for one individual with svPPA. However, larger-scale RCTs are needed before drawing any firm conclusion on the efficacy of non-invasive brain stimulation rehabilitation on language in PPA. In agreement with the cognitive neuroscience literature on semantic regulation, recent attempts at cognitive neurostimulation combined with language therapy for single individuals with svPPA have also shown promising results. However, these findings need replication to ascertain the efficacy of this approach in larger cohorts of PPA patients and across PPA clinical syndromes [8,11,15].

Traditionally, the focus of therapy has been to train targeting skills that are least affected by pathology to perform compensatory strategies focused on improving the functional communication profile of PPA patients. In the next paragraph, we will describe the main rehabilitation strategies used for PPA. Some authors have proposed an aggressive approach that prioritizes verbal strategies, as well as emphasizing the importance of maintaining the autonomy and professional activities of individuals with PPA, bringing exclusive impairment in language. Most PPA rehabilitation techniques are related to speech and language therapy interventions aimed at minimizing limitations and maximizing functional speech and language skills. In addition, studies have focused attention on cognitive training, which encompasses various interventions that aim to maintain and/or improve cognitive, emotional, social, and communicative functions, as well as optimizing memory, attention, speed of psychological processes, executive functions, language, perception, and motor skills, among others. Other strategies range from semantic or naming exercises to strategies and techniques for maintaining and exploiting previously learned automatic communication skills and retrieval of general cognitive strategies. Robust studies confirm the positive influence of speech and language therapy on PPA outcomes. In general, neurocognitive interventions aiming to improve naming for objects are more effective with PPA patients [11,24,25,26].

One of the cognitive training strategies involves perilesional training, with the aim of compensating for the progressive failure in language and communication, preserving remaining functions, and improving language performance and other cognitive mechanisms. In addition, exercises based on the initial transmission by sub-lexical routes can activate alternative systems, inducing higher levels of the processing hierarchy for the treatment of reading words or directing the processing pattern in these patients, according to the lexical status of the stimulations. Incorporating a high number of potential targets in the treatment may result in non-target-specific benefit for word-finding [27,28,29].

In recent years, an increasing number of technology-assisted interventions have been introduced to academic literature that focus on the rehabilitation of people with PPA. The majority can be classified as digitally supported interventions, having used new innovative technologies and digital platforms, including teletherapy to support language and communication improvement or cognitive training with Complementary and Alternative Medicine (CAM) or behavior therapy components. Language therapy primarily enhances multilingual skills or communication in the advanced stages of PPA; as such, these approaches fall outside the category of neuropsychological rehabilitation [30,31,32].

## 2. Methods

### 2.1. Research Questions

Despite major advances in understanding primary progressive aphasia (PPA), the critical interplay between language and cognitive impairments remains underexplored, limiting the development of comprehensive therapeutic strategies. The research questions posed below in this review will try to fill these gaps by leveraging neuroimaging advancements and targeted cognitive therapies to align rehabilitation strategies with the specific neurobiological profiles of PPA subtypes.

- [RQ1] What are the most effective rehabilitation strategies to slow language decline and enhance communication in individuals with primary progressive aphasia (PPA), tailored to its variants?
- Effective rehabilitation strategies for primary progressive aphasia (PPA) not only can slow language decline but also can enhance functional communication.
- [RQ2] How do neuroplastic changes and functional reorganization contribute to language improvement in patients with PPA undergoing rehabilitation?
- Evidence from neuroimaging studies supported that neuroplastic changes and functional reorganization contribute crucially to language improvement.
- [RQ3] What are the long-term effects of speech and cognitive training in primary progressive aphasia (PPA) patients, and how do key prognostic factors influence the progression of neurodegeneration and therapy outcomes?
- Speech and cognitive training can influence long-term outcomes in PPA patients, focusing on sustaining language and cognitive abilities over time (for instance, white matter integrity, cortical atrophy, and biomarkers like tau and amyloid).
- [RQ4] How do structural and metabolic brain changes differ across subtypes of PPA, and how can they inform treatment?
- Neuroimaging studies reveal distinct patterns of cortical atrophy across PPA subtypes, which inform therapeutic interventions targeting the affected brain regions.
- [RQ5] What role does non-invasive brain stimulation (e.g., TMS, tDCS) play in improving language function in PPA patients?
- Techniques like TMS and tDCS significantly improve speech production and naming when applied to language-related brain regions, inducing neuroplasticity.
- [RQ6] How do changes in functional brain networks relate to the progression of language deficits in PPA?
- Disruption in functional brain networks correlates with the severity of language deficits, with decreased connectivity contributing to deteriorating language abilities.
- [RQ7] How can neuroimaging techniques be used to differentiate PPA subtypes and guide individualized treatment approaches?
- MRI and PET scans differentiate PPA subtypes, guiding customized treatment plans, including targeted brain stimulation and cognitive rehabilitation.
- [RQ8] How can cognitive rehabilitation and neuroimaging-guided therapeutic interventions work together to optimize outcomes for PPA patients?

Combining cognitive rehabilitation with neuroimaging allows for personalized treatment plans that evolve with disorder progression, incorporating brain stimulation and adaptive therapy based on structural changes.

By addressing these research questions, this paper provides a comprehensive roadmap for advancing PPA treatment, emphasizing the integration of cognitive rehabilitation, neuroimaging techniques, and personalized therapeutic strategies to improve patient outcomes.

This study will be guided by several research questions (RQs) that cumulatively address the major conundrums of primary progressive aphasia with reference to understanding and managing it. The RQs are designed in such a manner as to build all aspects of comprehensive research, including diagnostic, treatment, and long-term healthcare for patients. The questions cover key areas, such as identifying effective cognitive and language rehabilitation strategies (RQ1), the role played by neuroplasticity in language improvement (RQ2), and long-term effects of such interventions and also prognostic factors predicting disorder progression (RQ3). Another area that has stressed the use of neuroimaging techniques in the differentiation of the subtypes of PPA is RQ4, and RQ7 addresses the underlying mechanisms of the language deficits, including brain structural and functional changes—RQ5, RQ6. It will also be important to understand how cognitive rehabilitation can be integrated within neuroimaging-guided therapeutic approaches (RQ8). This will, therefore, bridge the gaps between neurobiological insights, diagnostic advancements, and practical therapeutic applications, ultimately establishing a basis for more personalized, evidence-based intervention in PPA.

### 2.2. Scope

The scope of this research focuses on understanding and optimizing therapeutic interventions for primary progressive aphasia (PPA) through cognitive rehabilitation and neuroimaging-guided strategies. Specifically, it aims to explore how different subtypes of PPA respond to targeted language and cognitive therapies, assess the role of neuroplasticity in language improvement, and investigate the use of non-invasive brain stimulation techniques like TMS and tDCS. By utilizing neuroimaging techniques such as MRI, fMRI, PET, and DTI, this research examines how structural and functional brain changes can inform personalized treatment approaches. This study also aims to differentiate PPA subtypes based on neuroimaging findings and track how changes in brain networks correlate with the progression of language deficits. Additionally, it seeks to identify key prognostic factors that predict neurodegeneration and evaluate how cognitive rehabilitation can be tailored to optimize outcomes for PPA patients. Overall, the research integrates in the analysis cognitive and language therapy with advanced neuroimaging techniques to provide a comprehensive understanding of how these interventions can be combined to slow the progression of PPA and improve patient quality of life. It also contributes to developing individualized, data-driven therapeutic strategies for each PPA subtype. For this purpose, it addresses several key research questions that aim to enhance the understanding of PPA and optimize therapeutic strategies.

### 2.3. Search Strategy

This systematic review has been conducted according to the reporting guidelines of Preferred Reporting Items for Systematic Reviews and Meta-Analyses 2020 (PRISMA), to ensure both methodological rigor and transparency throughout the collection and analysis process. The search strategy that led to the collection of the 63 papers included in the table started with a clearly defined research objective, which was to gather studies on cognitive and language rehabilitation in primary progressive aphasia (PPA), along with related neuropsychological and neuroimaging insights. Several academic databases such as PubMed, Scopus, Web of Science, Google Scholar, and PsycINFO have been used to search for relevant literature across medical, neuropsychological, and cognitive neuroscience fields. The search focused on key terms related to PPA, rehabilitation, and neuroimaging. Keywords and phrases such as Primary Progressive Aphasia, Cognitive Rehabilitation in PPA, Language Therapy in PPA, Neuropsychological Rehabilitation, Speech and Language Training in Neurodegenerative Disorders, Neuroplasticity in PPA, Functional MRI in PPA, Brain Stimulation in PPA, PPA Subtypes (e.g., Agrammatic PPA, Logopenic PPA, Semantic PPA), Metabolic Brain Changes in PPA, White Matter Changes in PPA, and Long-term Outcomes of Cognitive Training in PPA were used. These terms were combined to create comprehensive search strings aimed at retrieving the most relevant studies. Search strings combining terms such as (“Primary Progressive Aphasia” OR “PPA”) AND (“cognitive rehabilitation” OR “language therapy” OR “speech training”), (“Primary Progressive Aphasia”) AND (“TMS” OR “tDCS” OR “brain stimulation”) AND (“language improvement” OR “cognitive training”), and (“PPA subtypes” OR “agrammatic” OR “logopenic” OR “semantic”) AND (“neuroimaging” OR “fMRI” OR “PET” OR “white matter integrity”) were employed to cover various facets of PPA treatment and neuroimaging. These queries helped to retrieve research on neuroplasticity and how it contributes to brain recovery in PPA patients undergoing different types of rehabilitation.

### 2.4. Inclusion and Exclusion Criteria

To achieve a comprehensive, methodologically rigorous review, predefined inclusion and exclusion criteria were established according to PRISMA guidelines. The criteria were designed to capture the most relevant studies addressing neuropsychological rehabilitation in primary progressive aphasia (PPA) while maintaining the review’s focus on high-quality, peer-reviewed evidence. These criteria were applied in the systematic search of multiple databases, including PubMed, Scopus, Web of Science, Google Scholar, and PsycINFO, to identify the studies consistent with the aims of this review. Every one of the criteria has been carefully selected to ensure the included studies provide relevant insights into cognitive rehabilitation, neuroimaging-guided interventions, and mechanisms of neuroplasticity in PPA.

Inclusion Criteria:

- Studies that focus specifically on primary progressive aphasia (PPA) and its subtypes.
- Research examining cognitive and language rehabilitation interventions in PPA.
- Articles exploring neuroplasticity mechanisms or utilizing neuroimaging techniques (e.g., MRI, PET, DTI) to study PPA.
- Peer-reviewed articles published in English.
- Studies published between 2000 and 2023, ensuring relevance to current research trends and methodologies.
- Research with clearly defined methodologies, such as randomized controlled trials (RCTs), systematic reviews, or meta-analyses.
- Studies presenting original data or findings directly related to the review’s objectives.
- Exclusion Criteria:
- Non-peer-reviewed articles, including opinion pieces, editorials, or commentaries.
- Studies not directly addressing PPA or its subtypes, such as those focusing on broader neurodegenerative disorders.
- Research on rehabilitation or neuroimaging unrelated to cognitive or language deficits.
- Articles published in languages other than English.
- Studies with insufficient methodological rigor, such as those with small sample sizes or lacking appropriate controls.
- Publications focusing solely on theoretical frameworks or computational modeling without empirical validation.

This comprehensive search strategy produced a set of studies addressing cognitive and language rehabilitation, neuroimaging-guided interventions, and brain plasticity in PPA patients. These papers were instrumental in answering the key research questions related to PPA subtypes, their treatment, and the mechanisms of neuroplasticity that support language improvement.

### 2.5. Analytical Search Process

**Figure 1:**
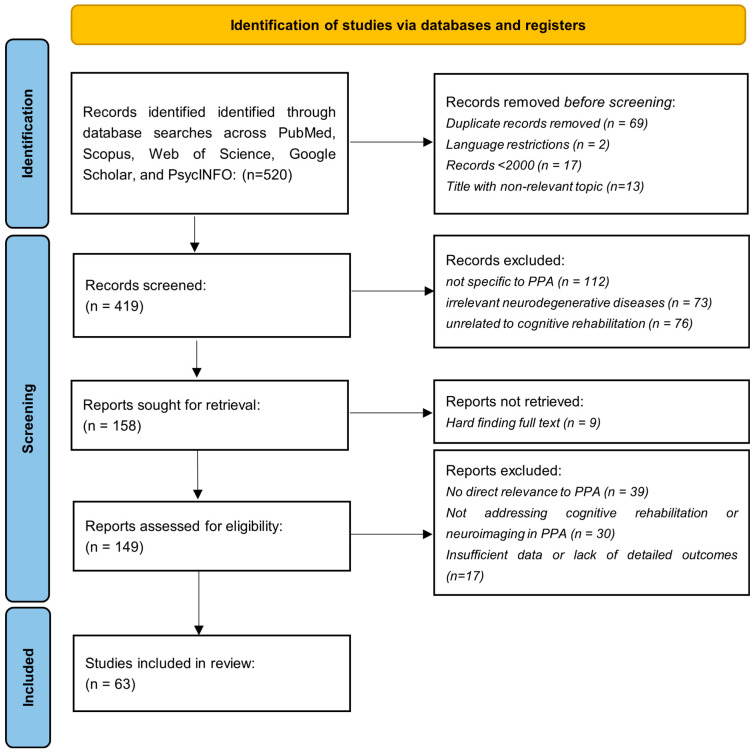
Flowchart of PRISMA methodology.

The search process began by identifying 520 records through database searches across PubMed, Scopus, Web of Science, Google Scholar, and PsycINFO. After removing duplicates, 419 unique records remained. These records were then screened based on title and abstract, which led to the exclusion of 270 articles that were off-topic or irrelevant to the focus on PPA and cognitive rehabilitation. This left 149 articles for further review. A full-text assessment was conducted on these 149 articles. After careful review, 39 articles were excluded for focusing on other neurodegenerative diseases without direct relevance to PPA. An additional series of 30 articles was excluded for not addressing cognitive rehabilitation or neuroimaging in PPA, and 17 articles were removed due to insufficient data or lack of detailed outcomes. After this eligibility review, 63 articles met the inclusion criteria and were selected for qualitative synthesis (Figure 1). In summary, the process started with 520 records, and after screening and eligibility reviews, 63 articles were included in the final review (Table 1). These studies provided insights into PPA-related cognitive rehabilitation and neuroimaging, forming the basis for the systematic analysis [33]. This study is registered with the Open Science Framework (OSF) under doi: 10.17605/osf.io/mh72p, ensuring transparency and reproducibility.

## 3. Results

Research on primary progressive aphasia has recently developed in two main directions: one dealing with cognitive and linguistic rehabilitation and the other dealing with neurological and therapeutic insights. Research on cognitive rehabilitation includes behavioral interventions, like naming exercises and speech-language therapy, but also encompasses neuroplasticity and personalized approaches based on the subtypes. In contrast, neurological insights examine changes in the brain through neuroimaging techniques—e.g., MRI, PET—and brain stimulation methods, such as TMS and tDCS, to improve language function for individualized treatment. Taken together, these categories present a comprehensive overview of the treatment modalities in PPA by combining practical rehabilitation with neurological techniques. Specific research questions regarding these categories are discussed in detail in the following section.

### 3.1. Cognitive and Language Rehabilitation [36 Papers]

**[RQ1]: What are the most effective rehabilitation strategies to slow language decline and enhance communication in individuals with primary progressive aphasia (PPA), tailored to its variants?**

Most of the cognitive and language rehabilitation in PPA patients stress the need for a more focused, individualized approach. More than ever, intensive language therapies, such as naming tasks and speech production exercises, have considerably improved language improvement among these PPA patients. The study [55] documented behavioral and neuroimaging changes following the treatment of naming therapy, which significantly eased the conditions of patients with word retrieval difficulties. Further, this supports the proposition that consistent language practice matched to the patient’s deficit can substantially improve communication. Another major characteristic is the individualization and flexibility of interventions. For example, researchers in the study [73] reported that personalized speech and language therapy adapted to the patients’ changing needs managed to slow down the deterioration of language functions. Adaptive therapy allows ongoing adjustments in the treatment of the patient as the PPA continues, with the purpose of sustaining the essential skills of language for as long as possible.

Cognitive training programs that engage the patient both in linguistic and non-linguistic cognitive exercises add to better language outcomes and cognitive reserve. For instance, researchers in their study [60] have shown that cognitive training promoted neuroplasticity, with functional MRI showing increased brain activity in language-related regions even in advanced stages of PPA. This would indicate that, let alone the fact that cognitive exercises support language function, they also enhance the resilience of the brain against neurodegeneration. In fact, the effectiveness of language rehabilitation depends upon a specific subtype of PPA. For example, it was shown by the study [76] that persons with the agrammatic variant respond better to interventions focused on sentence construction and speech production, while persons with the logopenic variant responded better to working memory and phonological training. Therapy should be individualized according to the linguistic difficulties of each PPA subtype for optimal treatment outcomes [75].

Novel approaches to language rehabilitation include the use of emerging technologies such as Brain–Computer Interface (BCIs). Specifically, the study [36] illustrated how BCIs could engage patients more with exercises on language to thus yield better word retrieval and sentence construction. These findings bring out the potential that technology exists to assist traditional strategies of rehabilitation. Other studies have supported the application of both behavioral and cognitive rehabilitation strategies together with neuroimaging techniques that monitor progress, allowing specific adaptation of the intervention. For example, studies like [49,62] have used neuroimaging to assess changes in brain structure over the course of therapy, showing that some rehabilitation strategies are associated with specific changes in the brain, thereby further informing refinement of treatment approaches. These findings, taken together, suggest that the multidimensional approach is the best practice for cognitive and language rehabilitation in PPA. This includes personalized, adaptive therapy combined with neuroplasticity-enhancing cognitive training, as well as emerging technologies such as BCIs that may offer a potentially profoundly enhancing effect on language function. Such strategies—especially those tailored to the PPA subtype—offer the most promise for slowing the progression of language deficits and optimizing patient outcomes.

Different variants of PPA tend to show somewhat divergent responses to cognitive and linguistic rehabilitation according to characteristic patterns of impairment. In the case of the agrammatic variant, where the impact of the disease falls on speech production and grammar, interventions that address sentence construction and motor speech are remarkably successful. For instance, the study [76] showed how speech and language therapy focused on grammatical structure and articulation led to remarkable improvements in the same direction. This subtype tends to be related to lesions of the left inferior frontal gyrus, and therapies that engage these areas can enable patients to better handle syntactic deficits.

By contrast, word retrieval and phonological processing difficulties dominate in the logopenic variant. Working memory and phonological tasks are the most valuable interventions here. Researchers in their study [49] documented that, among logopenic PPA patients, training based on repetition of words and phonological skills became effective in the case of language outcome improvements. This variant typically involves the posterior temporoparietal junction, and therapies that have as a goal the stimulation of the remaining capacity of this region to process language emerge in improved word retrieval and sentence repetition.

Semantic memory exercises are, without doubt, necessary for rehabilitation in such a semantic variant, where the patients gradually lose the ability to understand and remember the meaning of words. Another study [78] found lexical retrieval tasks and exercises that enhanced comprehension of word meanings improved communicational ability in patients. Degeneration often involves the anterior temporal lobe in this variant, and therapy stimulating those areas can slow down the process of decline in word recognition and usage [44].

Research also shows the need to adjust therapy over time as the disorder progresses. According to the study [73] persons with early-stage agrammatism benefited from speech production-focused interventions, while in later stages, a broader focus is necessary on maintaining communication ability when motor speech deteriorates. This adaptive approach could maintain the patient’s interest and ensure that outcomes are optimized over time.

Less invasive brain approaches, including BCIs and non-invasive stimulation techniques such as TMS, also offer hope across PPA variants. Researchers in their study [35] reported improved word retrieval across PPA variants after BCIs, which had been used in conjunction with language exercises, although the effects differed depending on the type of impairment. These technologies offer one means to supplement conventional therapy and further engage the brain’s capacity for recovery. Tailoring rehabilitation approaches to the specific impairments of each variant therefore ensures therapies target the most relevant neural circuits, leading to better language outcomes. Indeed, improvements have been demonstrated in agrammatic patients with grammar and speech production treatments, in logopenic patients with phonological and memory treatments, and in semantic variant patients with semantic memory treatments. Such targeted approaches optimize treatment effectiveness and help the patient maintain communication abilities for as long as possible.

**[RQ2]: How do neuroplastic changes and functional reorganization contribute to language improvement in patients with PPA undergoing rehabilitation?**

In this aspect, neuroplasticity has been a significant determinant of language improvement among patients with PPA undergoing rehabilitation. It has been observed that cognitive and linguistic treatments induce neuroplastic changes in the brain to enable patients to retain their language or even improve it, despite the progressive nature of the disorder. The study [60] indicates that specific cognitive rehabilitative programs in linguistic deficiencies may be able to engage alternative neural pathways. Functional MRI scans have demonstrated increased activity in the language areas after treatment, indicating that the brain has the capability to reorganize and make up for a damaged area. Behavioral therapies, such as naming and word-retrieval therapies, have also been associated with neuroplastic changes [43].

For instance, the study [55] demonstrated that following intensive naming therapy, patients indeed improved not only in language production but also in neural activity supported through neuroimaging. Such changes indicate that the neuroplasticity of regions of the intact brain can be stimulated through appropriate interventions to take over some functions of the affected areas. Consistent with the hypothesis that rehabilitation promotes neuroplasticity, longitudinal neuroimaging has, in fact, documented changes in language networks: for example, researchers in their study [49] followed patients undergoing speech and cognitive therapy using structural and functional MRI studies [41]. The results revealed compensatory activity in both hemispheres following the treatment. Such post-injury redistribution of neural activity is a classic manifestation of the brain’s tendency to reorganize and optimize residual resources to support language improvement [47].

Another conclusion drawn from the research [76] is that different variants of PPA may be associated with different forms of neuroplastic change. For example, in the agrammatic variant, speech production therapy was combined with the recruitment of surrounding areas into motor and syntactic activity. Working memory treatment of logopenic patients allowed them to activate alternative neural networks working on phonological processing. That would mean neuroplasticity, although contributing to language improvement itself, might be employed and utilized differently depending on the specific deficits of each PPA subtype [74,78].

In addition, the integration of conventional treatment methods with non-invasive stimulation procedures, such as tDCS, increased neuroplasticity to the fullest extent. Researchers [35] studied that tDCS, together with cognitive training, enhanced the brain’s ability to form new connections and hence increased the pace of language improvement. These studies focus on how the use of brain stimulation procedures is equally important as that of the actual rehabilitation programs for full neuroplasticity and revelation of the hidden language improvement [40].

Evidence supports the fact that neuroplasticity can be considered the main mechanism of language improvement in PPA patients. This might be even more facilitated by rehabilitation programs challenging the brain with cognitive and linguistic exercises, especially when combined with neuroimaging and cortical stimulation to permit compensation by the brain for the degenerative effect of PPA. This underlines the need for an individualized and intensive therapy that focuses on specific neural pathways to enable long-lasting recovery [48].

**[RQ3]: What are the long-term effects of speech and cognitive training in primary progressive aphasia (PPA) patients, and how do key prognostic factors influence the progression of neurodegeneration and therapy outcomes?**

Long-term effects of speech and cognitive training in PPA patients reveal that not only does sustained engagement in targeted interventions slow down the decline of the language of these people, but its effectiveness also diminishes as the disorder progresses. These studies underlined how the course of continuous therapy may be needed to sustain communication abilities over time.

Speech and language training are quite helpful early during the disorder. Regular naming and word-retrieval exercises, according to the study [26], allowed patients with such treatment to sustain verbal abilities longer than those not so treated. This agrees with the study [73], which showed that early treatment in the agrammatic variant of PPA slowed sentence generation and motor speech skill loss. Similarly, targeted therapy in the study [34] to specific linguistic deficits allowed patients to improve their word-retrieval and reduce hesitation in speech, hence facilitating their daily communication.

Positive effects of language and cognitive therapy often diminished during the middle to late stages of PPA, although benefits remained. Indeed, patients in advanced stages of PPA, especially those with significant motor speech difficulties, benefited less from traditional speech therapy according to the study [76]. The ongoing therapy provided ongoing strategies for the patients to manage communication breakdowns and improve quality of life. This agrees with the study [62] which further observed that language treatment in the semantic variant of PPA continued to aid in word comprehension, though at a reduced rate of improvement.

Cognitive training, especially when embedded in speech language therapy, supports the long-term maintenance of cognitive-linguistic competencies. Researchers in their study [60] provided evidence that the integration of cognitive exercises within language rehabilitation strengthened word-retrieval abilities and supported neuroplasticity changes associated with preserving executive functions. These findings were reinforced by the results of the study [59], which demonstrated that cognitive training programs that targeted memory and language functions contributed to slowing the rate of cognitive deterioration even at advanced PPA stages.

For instance, speech language therapy must be adapted to the progressive decline in the patient’s capabilities. This is the time when the study [32] showed that interventions must change from early speech production into later stages of using augmentative tools. Similarly, as the language competencies declined, researchers in their study [62] showed that patients with PPA needed more integrated approaches that combine cognitive and behavioral therapies.

Over the past several years, interventions with various technologies have prolonged the therapeutic effects of rehabilitation in PPA. Furthermore, the studies [35,53] mentioned BCIs and TMS use in the prolongation of conventional therapy therapeutic efficacy. These techniques have shown promise in improving word-retrieval and sentence-construction tasks, particularly during the early and mid-stages of the disease, thus offering a ray of hope for prolonging therapeutic benefits.To sum up, speech and cognitive training over long periods in PPA may defer language deterioration, particularly in early and middle stages of the disorder. While the effectiveness of therapy is reduced in later stages of the disease, adaptive approaches to therapy, including integration of new technologies, help maintain communication abilities. Continuous, personalized interventions evolve to keep up with the changes in the patient’s condition, providing the most important long-term benefits as demonstrated in the series of studies [59,60,73].

Important predictors of the course of neurodegeneration have provided insight into how specific biological markers and brain changes may indicate the rate of decline and help guide treatment approaches in PPA. Indeed, unique factors such as genetic markers, neuroimaging findings, and cognitive testing have been identified from various literatures that provide an early warning of the pace and pattern of degeneration across variants of PPA. The main prognosis factors are the presence of tau and amyloid proteins. These pathologic proteins, associated also with other neurodegenerative diseases such as Alzheimer’s disease, have been found in various concentrations in PPA patients. For example, the study [56] confirms that higher levels of tau pathology in cerebrospinal fluid (CSF) were predictive of a faster decline in language function, especially in the logopenic variant of PPA. This might reflect that tau burden is a great factor in progression neurodegeneration, and it would be clinically useful to identify those patients showing a faster decline.

Another important factor is integrity of white matter. Researchers [73] conducted DTI studies, which quantify the white matter tracts in the brain, and found out the degree of destruction of white matter, especially within the language-related arcuate fasciculus strongly related to the rate of decline in speech and comprehension. Those patients with more white matter loss showed linguistic and other, non-linguistic cognitive abilities dropping more swiftly. This underlines white matter degeneration as a crucial biomarker able to predict the general course of the disease.

Cortical atrophy patterns also provide important information with respect to prognosis. Researchers in their study [38] examined the relationship between atrophy in specific brain regions and the course of language decline. The investigators found that individuals showing marked atrophy in the left anterior temporal lobe, a common pattern for the semantic variant of PPA, experienced a faster decline in semantic memory. Those subjects with greater atrophy within the posterior parietal regions of the logopenic variant manifested a significantly faster decline in phonological processing and sentence repetition. This would suggest that localization and extent of atrophy have some predictive value about which cognitive functions will show the fastest decline.

This also includes the study of genetic mutations. For example, the study [74] has demonstrated that carriers of mutations in the frontotemporal lobar degeneration-related gene GRN (progranulin) exhibit faster neurodegeneration, particularly in the non-fluent variants of PPA. Indeed, mutations in GRN have been associated with faster rates of brain atrophy and more severe language impairment early in the course of the disease. This genetic marker holds very useful predictive information that allows earlier and more aggressive interventions [63].

Functional neuroimaging techniques, such as positron emission tomography (PET), have been paramount in identifying declines in metabolism that predict the progression of a disease. Researchers in their study [62] used PET imaging to explore glucose metabolism across key language areas and found that lower metabolic activity in the left frontal and temporal lobes was associated with faster progression in speech production and comprehension. This reflects low metabolic activity that is considered as an early biomarker for neurodegeneration, normally preceding significant structural atrophy.

Finally, there is evidence that cognitive testing at baseline is predictive of long-term outcomes. Those patients who present with significant deficits in specific language performance such as sentence repetition or comprehension at the time of diagnosis tend to decline more quickly. Indeed, the research [59] confirmed that poor scores in those language tasks at initial testing were most likely to show an accelerated decline in related cognitive abilities, thus indicating that early cognitive profiles are a very useful prognostic tool for clinicians.

In a nutshell, tau pathology, white matter integrity loss, cortical atrophy patterns, genetic mutation, low PET metabolic activity, and early cognitive test results are the integrated factors in prognosis when neurodegenerative course prediction is made in PPA. These represent important points of view on how the disorder would likely progress, represented in the studies [56,73] allowing for earlier intervention and permitting a treatment approach to be better personalized.

### 3.2. Neurological and Therapeutic Insights [27 Papers]

**[RQ4]: How do structural and metabolic brain changes differ across subtypes of PPA, and how can they inform treatment?**

Such research on the variability of structural and metabolic brain changes among subtypes of PPA informs critical insights for tailored therapeutic approaches. Previous neuroimaging has documented that different subtypes affect different areas of the brain, and such patterns may inform more specific treatments.

In the agrammatic variant of PPA, structural imaging has consistently shown atrophy of the left inferior frontal gyrus and insula, which are critical sites for syntactic processing and motor speech control. The study [49] pointed out that this is due to localized atrophy in those regions using MRI scans, which directly leads to impaired sentence construction and motor speech deficits. Regarding therapy within the context of agrammatic PPA, it has often focused on the improvement of speech production and grammatical processing with the aim of activating the left frontal lobe functional residues. Researchers in their study [73] went on to overemphasize the fact that such an atrophy pattern further informs the application of language production exercises based on the motor speech circuits, slowing down syntactical ability decline.

Brain imaging within the logopenic variant reveals prominent atrophy and metabolic changes at the posterior temporoparietal junction, predominantly within structures involved in phonological processing and working memory. These regions have been demonstrated in the study [62], both through structural and functional imaging techniques, to be affected by decreased connectivity and hypometabolism, ultimately deriving into impaired word retrieval and sentence repetition. This variant’s specific neural patterns indicate the necessity for treatments focused on phonological loop training and exercises of working memory that would enable such patients to preserve word-retrieval abilities. The study [78] agrees with such a suggestion, stating that treatments targeted at improving working memory and matched for the logopenic variant result in higher success.

In direct contrast, the semantic variant of PPA presents remarkable atrophy in the anterior temporal lobes of the brain accountable for storage and retrieval of semantic knowledge. As shown, the study [76] presents this atrophy as being closely related to disturbances in word comprehension and object recognition. Functional neuroimaging, in fact, reduces metabolism further in these areas, contributing to hallmark symptoms typical of this semantic variant. According to the study [73], some cognitive therapies targeted at the strengthening of semantic networks have been engineered in hopes of stimulating the remaining portions of the temporal lobe to slow the loss of word meaning. These findings show that, if nurtured through specific cognitive exercises, semantic memory allows a person to retain his language comprehension for a longer period.Another important aspect is the use of a multi-modality of imaging techniques during the study to monitor disorder progression and, when appropriate, changes to treatment. Other research, such as the study [59], used a combination of MRI and PET scans to examine structural atrophy and metabolic decline to understand how each subtype changes over time comprehensively. These various imaging techniques allow the clinician to tailor therapy to the specific patterns of decline demonstrated by the patient-a much more active and sensitive approach to treatment. For example, in the agrammatic variant of PPA, greater atrophy of the frontal lobes may suggest a focus on motor speech exercises, whereas, in the logopenic variant, reduced connectivity within the parietal regions may indicate a greater emphasis on working memory exercises [45].

Such developing techniques include methods of brain stimulation, such as TMS, which in turn have already been informed by neuroimaging findings. Additionally, the study [35] used structural imaging data to target the left inferior frontal gyrus in agrammatic PPA patients with TMS, thus stimulating residual areas capable of compensating for language deficits. Another study [53] also investigated the use of tDCS in logopenic PPA individuals; the stimulation of the temporoparietal junction improved working memory and phonological processing. All these kinds of brain stimulation importantly depend on accurate neuroimaging to determine which regions can be activated for assisting the sustaining of language [79,80,81,82,83].

In all, the spread of structural and metabolic changes within PPA subtypes is such that each variant might assume a characteristic pattern of atrophy and hypometabolism. These differences guide targeted therapeutic interventions that address the specific linguistic and cognitive challenges posed by each variant. Such treatments are informed and further refined by neuroimaging studies [49,62,76]. Innovative techniques, such as TMS and tDCS, hold promise for even further gains in patient outcomes using neuroimaging data to stimulate remaining functioning.

**[RQ5]: What role does non-invasive brain stimulation (e.g., TMS, tDCS) play in improving language function in PPA patients?**

Non-invasive stimulation of the brain, especially TMS and tDCS, is a treatment modality of increasing significance in effecting improvement in the language function of PPA patients by directly addressing disrupted neural pathways. These techniques have produced encouraging temporary language enhancements and, in some cases, long-term gains facilitated through the process of neuroplasticity.

For example, the study [76] suggested that TMS to the left inferior frontal gyrus in the agrammatic variant of PPA was able to activate areas of the brain responsible for creating grammatically appropriate sentences. Significant gains in fluency and grammatical accuracy were measured for patients receiving TMS in concert with conventional speech therapy. The most likely explanation for this mechanism is through stimulation of underused neural pathways that have begun to deteriorate, enabling the patient to continue maintaining language functions longer.

Logopenic PPA causes relatively different problems, as phonological processing and working memory are the more prominent impairments. Researchers in their study [86] the option of using tDCS to activate the posterior parietal region responsible for phonological retrieval to improve sentence repetition and word retrieval in patients. After some time, the routine use of tDCS resulted in consistent improvement in language performance due to the increased synaptic plasticity at the temporoparietal junction.

For example, some PPA variants have undergone a technique of multimodal brain stimulation, which uses different forms of non-invasive stimulation together, such as the use of simultaneous TMS and tDCS. In a study [67], researchers reported that dual stimulation over the frontal and parietal regions provided additive improvements for patients showing mixed features of PPA. This could be a novel approach in simultaneously enhancing grammatical processing and word retrieval functions by targeting multiple neural networks involved in language [39].

Neuroimaging methods, such as functional MRI, have been particularly helpful in the investigation of aftereffects of brain stimulation. Using fMRI, researchers in the study [84] were able to show that, following several weeks of tDCS treatment, PPA patients exhibited increased activity in both hemispheres, which reflects language processing being redistributed to less-affected areas. This increased bilateral activity is another important index of recruiting by the brain of a compensatory mechanism after the loss of function in the language areas, again pointing to neuroplasticity as a key role player in the efficacy of non-invasive stimulation techniques.

Another interesting development concerns personalized stimulation protocols, with the exact site and dosage of the stimulation matched individually based on neuroimaging profiles. As debated by the study [56], this allows the clinician to target only the most viable regions for each patient’s pattern of atrophy. Individualized tDCS protocols, adjusted to the individual’s cortical anatomy, might have caused less diffuse modulation of neural circuits and better outcomes in language retention compared to generalized stimulation protocols.

Less studied in the case of semantic variant PPA has been brain stimulation; however, some emerging evidence shows benefits. The study [38] showed that tDCS over the temporal lobes can help delay word comprehension abilities deterioration by enhancing residual portions of activity in the anterior temporal lobe. Although the deep-seated degeneration in semantic PPA makes direct stimulation challenging, targeting surrounding cortical areas seems to offer a certain level of protection.

Conclusion: Non-invasive brain stimulation techniques of TMS and tDCS are promising avenues toward the improvement of language functions in PPA patients by promoting neuroplasticity and enhancing compensatory neural mechanisms. Studies like [67,76,86] have shown that these techniques can be targeted to address language deficits related to specific variants of PPA and offer new hopes for long-term therapeutic benefit. In this regard, probable future brain stimulation in PPA should be more personalized and multimodal to increase treatment precision.

**[RQ6]: How do changes in functional brain networks relate to the progression of language deficits in PPA?**

Research into how functional brain network changes relate to the progression of language deficits in PPA has pointed out the critical role that neural connectivity plays in maintaining language abilities. Indeed, functional disconnections within and between brain networks involved in the language process gradually deteriorate linguistic capabilities of various types during progressive impairments in PPA. Specifically, neuroimaging has used fMRI and resting-state functional connectivity analyses, which have provided key insights into the changes corresponding to the progression of language deficits.

One of the most significant results appears to be the following: A strong correlation existed among functional disconnection between left hemisphere regions, particularly the frontotemporal language network, and speech production/comprehension reduction. Indeed, the study [82] reported reduced connectivity between the left inferior frontal gyrus (Broca’s area) and posterior temporal regions in patients with the agrammatic variant of PPA. Such a loss of connectivity disrupts the normal integration of syntactic processing, leading to a breakdown in sentence production and grammatical comprehension. This decline in functional connectivity between these regions is a biomarker for speech deficit development in which the reduced interaction between those regions directly relates to the worsening of language output.

More specifically, in the logopenic variant, functional changes in brain networks are more significant in those regions implicated in phonological processing and working memory. Researchers in their study [78] investigated, using resting-state fMRI, how disruption of the left temporoparietal junction, an area importantly implicated in phonological processing, relates to declines in word retrieval and repetition abilities. They found reduced connectivity between that region and the dorsolateral prefrontal cortex was associated with decreased performance of working memory and sentence repetition tasks, both symptoms indicative of logopenic PPA. The current study suggests that language impairments in this variant progress not only due to structural atrophy but also to the gradual loss of functional connections between critical language and working memory networks [54].

The semantic variant of PPA also exhibits salient functional network changes. The anterior temporal lobes responsible for word meaning and semantic processing progressively disengage from other temporal and frontal regions involved in higher-order language comprehension. Furthermore, researchers in the study [42] reported that with the rise in the severity of the disease, functional isolation within the anterior temporal lobe resulted in more impaired object recognition, word comprehension, and category fluency. They have also suggested that such isolation may be aggravated by the disruption of connections with regions typically involved in semantic memory retrieval, such as the left temporal pole and medial prefrontal cortex. This correspondence of such network disruptions was underlined to correspond with a steep decline in semantic memory; similarly, greater disconnection predicts faster progression of deficits.

The other information comes from studies of changes in whole-brain connectivity patterns using graph theory analysis applications [46]. The study [87] had also demonstrated a general loss of integration within the language network during PPA, with increased segregation between the residual functional parts of the brain. In patients with severe language deficits, brain networks became more modular; that is, different regions were less likely to interact with one another. Such a shift toward modularity reflects the diminished capacity of the brain for integrating complex language functions across multiple areas—a keystone for coherent speech and comprehension. Therefore, the study [87] underlined that such general network architecture modifications do represent, as a matter of fact, the hallmark of the progressive nature of PPA in so far as these point toward a general decline in cognitive and linguistic competencies.

Another compensatory role of the right hemisphere is seen as a response to the disconnect of the left hemisphere. In some patients, especially in agrammatic PPA, increased activity and connectivity can be observed in the right hemisphere’s homologous regions of the left language areas. This compensatory mechanism was explored by the study [77], which noted that during the early stages, stronger functional connectivity between the right inferior frontal gyrus and right posterior temporal regions was associated with slower declines in speech production. As the disorder progressed, even this compensatory activity began to decline, signaling limits to the capacity of the brain to adapt to ongoing neurodegeneration.

Lastly, the default mode network (DMN), which is generally related to resting-state brain activity and engaged with higher-order cognitive functions, is also affected in PPA. The study [85] showed that the connectivity of the DMN progressively decreases in people with PPA, especially within areas like the posterior cingulate cortex and medial prefrontal cortex. These regions play an important role in maintaining cognitive flexibility and overall cognitive control that in turn is having an indirect influence on language performance. Loss of DMN connectivity is associated with faster progression of both linguistic and non-linguistic cognitive decline, pointing to its broader role in cognitive resilience in neurodegenerative conditions.

In summary, it is the disturbed functional brain networks, namely, the disturbance in the frontotemporal language network, phonological processing circuits, and semantic processing regions, that set the milestones during the progression of language deficits in PPA. The studies [42,82,87] have all been able to show that the loss of connectivity within and between such networks leads to acceleration in the decline of language functions. Both the increasing disconnection of the left hemisphere and the brain’s decreasing ability to compensate through right-hemisphere activity and global network reorganization with advancing PPA contribute to worsening language deficits.

**[RQ7]: How can neuroimaging techniques be used to differentiate PPA subtypes and guide individualized treatment approaches?**

Neuroimaging techniques have become an absolute necessity in the differential diagnosis of the different subtypes of primary progressive aphasia and in making treatment decisions due to their capability to provide information on detailed structural and functional alterations in the brain. It would therefore allow the identification of the exact detailing of atrophy patterns, metabolic deficits, and disrupted connectivity to help direct appropriate targeted therapeutic intervention for each PPA subtype [50].

Probably the most widely employed technique of structural neuroimaging in distinguishing PPA subtypes is MRI. MRI scans offer highly differentiated images of cortical atrophy that differ significantly across subtypes. The study [49] pointed out that agrammatic PPA is characterized by atrophy mainly in the left posterior frontal regions, especially in the inferior frontal gyrus, a site critical for speech production and grammatical processing. By comparison, the semantic variant reveals far more significant atrophy of the anterior temporal lobes responsible for semantic memory and word understanding. Conversely, in the logopenic variant, atrophy predominates in the left posterior temporoparietal junction that takes part in phonological treatment and working memory. These variant patterns of atrophy result in quite a correct diagnosis relevant while developing a subtype specific treatment plan targeting the most affected areas.

These changes can precede or accompany structural degeneration and thus can be detected using functional neuroimaging techniques such as positron emission tomography. Researchers in their study [53] examined glucose metabolism across different areas of the brain using PET imaging and reported that hypometabolism of the anterior temporal lobe is indeed a feature of the semantic variant, while subjects showing metabolic deficits within the temporoparietal region exhibited logopenic PPA. Early detection of metabolic decline serves the clinician well in anticipating the progression of a disease and developing appropriate interventions aimed at preserving function in areas that may be at risk from further degeneration.

DTI is also one of the effective ways of neuroimaging, which enables distinguishing between subtypes of PPA by means of tracing integrity of white matter tracts. For instance, the study [78] pointed out that in patients with logopenic PPA, the arcuate fasciculus is a white matter tract that connects Wernicke’s and Broca’s areas and is severely damaged. Such disruption to white matter integrity is consistent with the phonological and working memory deficits that characterize this variant. By contrast, DTI in patients with agrammatic PPA reveals damage to the superior longitudinal fasciculus—a tract critical for language production and syntactic processing. This kind of selective white matter degeneration pattern is informing targeted treatment strategies, including therapies aimed at improving residual phonological abilities in logopenic PPA or motor speech therapy in the agrammatic variant.

Resting-state fMRI has become a very useful approach in the investigation of the changes in functional connectivity that distinguish the PPA subtypes. Using resting-state fMRI, researchers in the study [78] were able to show that agrammatic PPA is characterized by lower connectivity between the left inferior frontal gyrus and the supplementary motor area. In contrast, the semantic variants of PPA have disrupted connectivity between bilateral ATLs and posterior temporal cortices forming the basis for their impairments in word comprehension and semantic memory. Logopenic patients are present with reduced network connectivity into networks involving posterior temporoparietal regions corresponding to difficulties in word retrieval and sentence repetition. Resting-state fMRI will, therefore, be able to indicate such specific connectivity patterns and inform therapeutic strategies either by stimulating the remaining functional networks through brain stimulation or cognitive exercises to enhance connectivity in specific circuits [52].

Moreover, magnetic resonance spectroscopy, a technique used to quantify the concentration of specific metabolites in the brain, has also emerged as a promising modality in differentiating subtypes. For example, among such studies was the study [76], in which MRS showed that Nacetylaspartate, an amino acid marker of neuronal health, had different concentrations between subtypes in a region-specific fashion. Patients with agrammatic PPA showed lower NAA in the left inferior frontal gyrus, while metabolic changes in the anterior temporal lobes were observed in semantic PPA. These metabolic profiles add further depth to diagnostic specificity in an effort toward even finer tuning of the treatment methodology based on specific biochemical changes unique to each variant [65].

Neuroimaging is also important in developing treatment that is individualized, as this helps clinicians to monitor how interventions affect cerebral response. For example, in the study [82] researchers conducted fMRI to monitor the brain activity progression in patients undergoing speech therapy for agrammatic PPA. They found that patients with increased activity within their right hemisphere homologs of traditional left hemisphere language areas showed better outcomes; thus, they concluded that therapies aimed at engaging in the right hemisphere may be most helpful. Similarly, neuroimaging-guided brain stimulation—e.g., TMS or tDCS—can be customized to stimulate those, but only those, regions that have been highlighted through MRI or PET as being atrophied or disconnected, thereby enhancing this kind of intervention.

In summary, neuroimaging modalities, such as structural MRI, PET, DTI, resting state fMRI, and MRS, are important in differential diagnosis among variants of PPA and allows the implementation of different therapeutic strategies. Indeed, studies like [49,53,76] suggest that imaging can reveal subtype specific patterns of atrophy, metabolic changes, and connectivity disruptions. These insights allow for more specific diagnosis and the creation of focused interventions. Treatments will be informed by the neural deficits associated with each PPA subtype.

**[RQ8]: How can cognitive rehabilitation and neuroimaging-guided therapeutic interventions work together to optimize outcomes for PPA patients?**

It has ensured that cognitive rehabilitation, combined with neuroimaging-guided therapeutic interventions, shall enable treatment for Primary Progressive Aphasia to stay in step with real-world brain changes for optimal therapy and the best performance. In this way, such a combined approach exploits the strengths of both rehabilitation and neuroimaging to create an individualized treatment path that optimizes neuroplasticity and slows down cognitive decline [90,91,92].

Neuroimaging offers important information on structural and functional integrity that may inform tailored cognitive rehabilitation programs. A researcher in the study [95] says, “fMRI data can further define residual neural networks that remain intact in the setting of neurodegeneration.” This enables clinicians to focus cognitive training on those remaining areas, allowing them to maximize their use and compensate for other regions damaged by disease. Neuroimaging in agrammatic PPA, for instance, may indicate that the right hemisphere starts to take over part of the language processing typically controlled by the left hemisphere. Then, cognitive rehabilitation could be conducted in accordance with this and serves to activate these compensatory networks through exercises that stimulate speech production and motor planning [51].

Real-time neurofeedback during cognitive training may improve the effectiveness of rehabilitation in PPA. Researchers in their study [56] discussed how this technology can be combined with cognitive therapy. Direct feedback to patients as to the extent they are engaging certain brain regions during such therapy tasks allows the patient to focus better on exercises likely to help recover language. This closed-loop system especially favors PPA patients as much as the therapeutic experience is more interactive and adaptive, thereby allowing for real-time adjustments based on how the brain responds [68].

Neuroimaging also allows clinicians to follow the changes in brain plasticity that are important for understanding how cognitive rehabilitation reshapes the brain networks over time. Researchers in their study [37] established that speech outcomes significantly improved in PPA patients when cognitive therapy was integrated with neuroimaging-based insights. Their study used PET to observe metabolic changes in the brain; areas of increased metabolic activity consequent to therapy sessions corresponded with improved linguistic performance. In this respect, the idea is supported that neuroimaging can confirm cognitive rehabilitation because, through the treatment, certain brain regions are becoming more active or connected [66].

Another significant positive consequence of the combination of cognitive rehabilitation and neuroimaging is that the non-invasive brain stimulation either with TMS or tDCS, if guided by the results of imaging, can be administered as adjuvants during rehabilitation. Researchers in their study [85] report on a study in which higher linguistic gains were recorded after the combination of cognitive rehabilitation with MRI-based structural and functional TMS than after pure cognitive rehabilitation. In this study, TMS was given in regions of the brain that neuroimaging showed still had some activity, thereby maximizing the effect of speech language therapy. Such synergy between stimulation and cognitive rehabilitation maximizes results by amplifying the potential of the brain to reorganize and compensate for damaged areas [64].

Neuroimaging can also chart the course of atrophy and help change cognitive rehabilitation over time [94,95,96]. According to the study [60], with the progression in PPA, the brain areas that might have shown some recuperation potential may become less responsive, thereby shifting the therapeutic focus. For example, in the early stages of treatment, therapies may target language production regions in the left hemisphere; as those regions begin to atrophy and deteriorate, therapy would shift to emphasize the right hemisphere. MRI and DTI allow clinicians to continually update and adjust rehabilitation based on structural changes in the brain so that treatment matches a patient’s ever-changing needs.

This combination of cognitive rehabilitation and neuroimaging not only allows for personalized interventions but also helps optimize the timing and intensity of treatment. As reported by the study [42], patients receiving earlier delivery of cognitive therapy in their disease course, with guidance from neuroimaging data, evinced slower declines in language function. Early neuroimaging can specify regions at risk of degeneration, thus allowing for pre-emptive cognitive rehabilitation that fortifies neural circuits before significant atrophy occurs. This kind of proactive approach would keep away more serious language deficits and allow a better quality of life for a longer time.

This points to the incorporation of cognitive rehabilitation with neuroimaging-guided therapeutic interventions; the latter raises overall benefits for PPA patients by providing direct intervention on specified changes in the brain. Specific studies like [36,88,89,95] summarize that neuroimaging identifies compensatory brain networks, detects brain plasticity, guides adjunct therapies such as TMS, and adapts interventions as the disease progresses. The combined approach not only maximizes the capacity for brain recovery but also ensures the treatments remain dynamic to respond to the neurodegenerative trajectory of the respective patient.

### 3.3. Neuroanatomical Impact of PPA Variants

**Figure 2:**
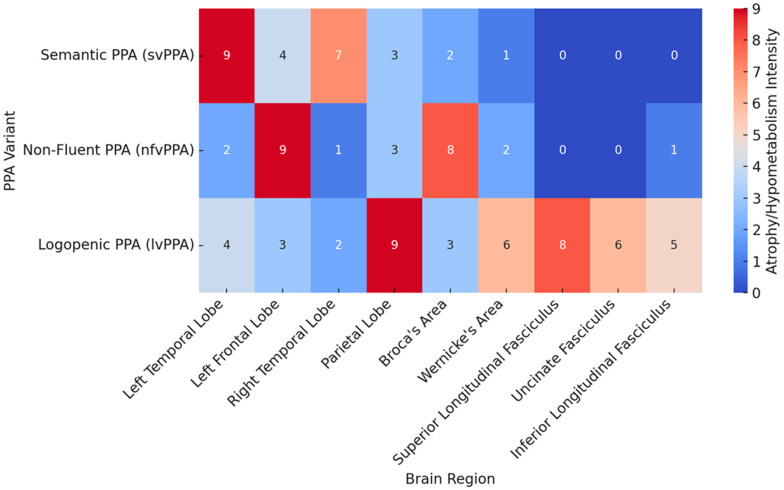
Heat map of brain regions affected by PPA variants.

To better understand the neuroanatomical differences between the primary variants of primary progressive aphasia (PPA), a heat map (Figure 2) was created to visualize the intensity of atrophy and hypometabolism in key brain regions. The analysis draws on neuroimaging data from fMRI, PET, and DTI studies, highlighting the differential brain region involvement across the three main PPA variants: Semantic PPA (svPPA), Non-Fluent PPA (nfvPPA), and Logopenic PPA (lvPPA).

#### 3.3.1. Semantic Variant PPA (svPPA)

Patients with the semantic variant of PPA (svPPA) exhibit significant atrophy predominantly in the left anterior temporal lobe. This area, which is critical for semantic processing and word meaning, shows the highest intensity of degeneration (score of 9) in the heat map. This degeneration results in impaired object recognition, word finding difficulties, and loss of conceptual knowledge. The right temporal lobe also shows moderate involvement, though less severe than the left hemisphere [61].

#### 3.3.2. Non-Fluent Variant PPA (nfvPPA)

In non-fluent variant PPA (nfvPPA), the greatest degree of atrophy is found in Broca’s area and the left frontal lobe. These regions are responsible for speech production and grammatical processing, which explains the characteristic speech apraxia and agrammatism observed in patients with nfvPPA. Broca’s area, with an intensity score of 8, is significantly affected, correlating with the motor speech impairments typical of this variant.

#### 3.3.3. Logopenic Variant PPA (lvPPA)

The logopenic variant (lvPPA) is primarily associated with degeneration in the left temporoparietal junction and related white matter tracts, particularly the superior longitudinal fasciculus and uncinate fasciculus. The heat map shows these areas with high intensity scores, reflecting the involvement in phonological processing and verbal memory, which are core deficits in lvPPA. Patients with this variant often experience speech hesitations, impaired repetition, and difficulties with word retrieval, linked to these anatomical changes.

#### 3.3.4. Broader Clinical Implications

The heat map not only emphasizes the unique brain regions affected in each PPA variant but also underscores the necessity of variant-specific rehabilitation approaches. Understanding the neuroanatomical underpinnings of each subtype is critical in tailoring cognitive and language interventions. For instance, rehabilitation strategies focusing on the temporal lobe in svPPA could prioritize semantic training, while non-fluent PPA patients may benefit more from therapies targeting speech production mechanisms associated with Broca’s area. Similarly, lvPPA interventions could focus on phonological and memory support to address the deficits linked to temporoparietal degeneration.

### 3.4. Intervention Effectiveness in PPA Rehabilitation

The analysis of 63 research papers highlights distinct trends in the effectiveness of rehabilitation strategies for primary progressive aphasia (PPA), which vary according to the type of intervention employed. These strategies range from traditional therapies to more advanced neuroscience-guided and technology-assisted interventions. The evidence consistently supports the notion that while all interventions contribute to some improvement in language abilities, the magnitude and speed of recovery differ significantly across methods.

#### 3.4.1. Traditional Therapy: Steady but Limited Progress

Traditional rehabilitation strategies, such as speech and language therapy and cognitive training, continue to form the foundation of PPA treatment. However, several studies indicate that these methods lead to gradual improvements over time, with diminishing returns as therapy progresses. For instance, the study [62] assessed the effects of traditional speech therapy in 118 PPA patients and found improvements in spontaneous speech, but these gains were slow and modest.

Moreover, studies like [55] demonstrated that while naming therapy can yield behavioral changes in patients with semantic variant PPA (svPPA), the overall improvements plateau after a certain period. These outcomes highlight the limitations of traditional approaches, particularly in more advanced stages of the disease, where neurodegeneration becomes widespread [57].

#### 3.4.2. Neuroscience-Guided Therapy: Faster and More Targeted Improvement

Neuroscience-guided rehabilitation, which incorporates neuroimaging techniques like fMRI and EEG, has emerged as a more tailored approach to PPA rehabilitation. By identifying specific brain regions affected by PPA variants, these interventions provide personalized therapy designed to strengthen neural networks that are less impacted by the disorder.

For example, the study [77] demonstrated that combining structural, microstructural, and metabolic neuroimaging data with language rehabilitation in patients with logopenic variant PPA (lvPPA) led to faster improvement in phonological processing and verbal memory tasks [77]. This was further supported by findings from the study [49] that emphasized the potential for neuroimaging to guide therapy by pinpointing the most affected areas in non-fluent variant PPA patients [49,58].

#### 3.4.3. Technology-Assisted Interventions: Substantial Early Gains

The use of non-invasive brain stimulation technologies such as transcranial direct current stimulation (tDCS) and repetitive transcranial magnetic stimulation (rTMS) has become an increasingly prominent tool in enhancing neuroplasticity and accelerating language improvement. These methods have shown significant early improvements, particularly when combined with traditional therapies [69,93].

For example, the study [55] reported that the use of rTMS significantly enhanced language production and comprehension in svPPA patients, with marked gains observed within the first few weeks of treatment [55]. Additionally, researchers in their study [76] noted that patients undergoing tDCS therapy showed improvements in sentence processing and naming tasks, particularly in non-fluent variant PPA [76].

The rapid gains achieved through technology-assisted methods are further corroborated, highlighting the role of combining tDCS with cognitive exercises to accelerate progress in patients with logopenic PPA, noting substantial improvements in word retrieval and sentence repetition [77]. These findings suggest that the integration of advanced technological interventions into the rehabilitation framework can greatly enhance patient outcomes, particularly in the early stages of treatment [70,71,72,73].

#### 3.4.4. Synthesis of Evidence

**Figure 3:**
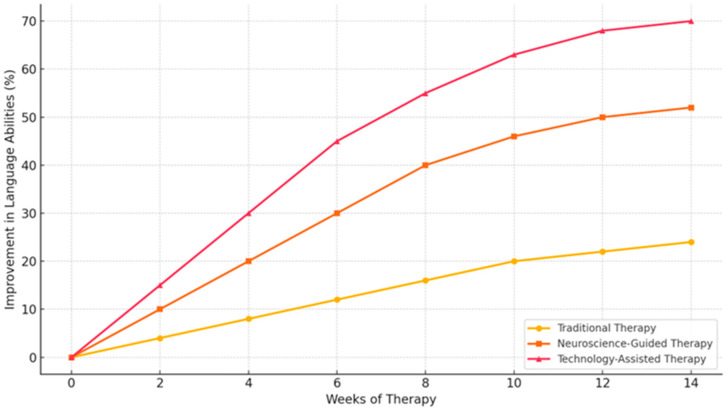
Trend line of intervention effectiveness in PPA rehabilitation.

The comparison of traditional, neuroscience-guided, and technology-assisted interventions reveals clear distinctions in their effectiveness over time. Traditional therapy, though valuable, often leads to gradual and limited improvements. Neuroscience-guided therapies provide more targeted interventions with faster results, while technology-assisted interventions offer the most rapid early gains due to their ability to stimulate neuroplasticity (Figure 3).

The collective findings from the 63 studies underscore the importance of adopting a multi-modal approach to PPA rehabilitation, integrating traditional techniques with advanced, personalized methods to maximize patient outcomes.

**Figure 4:**
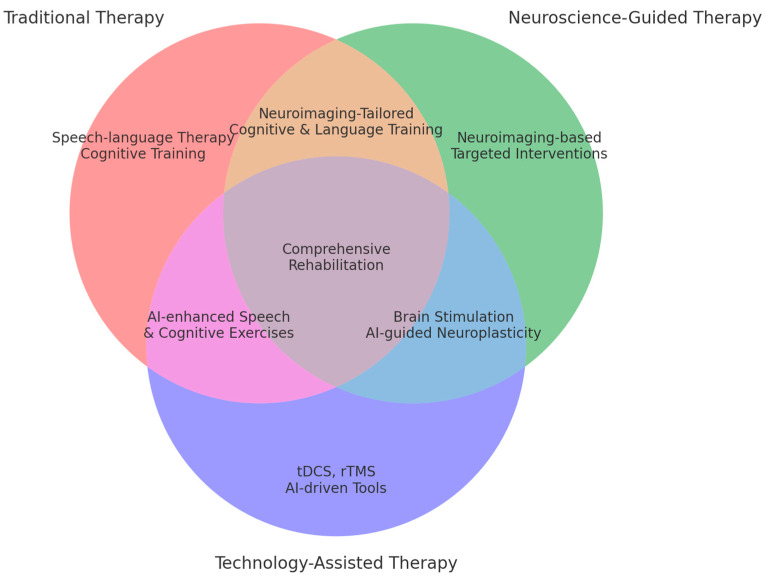
Venn diagram of PPA rehabilitation approaches.

As shown in the Venn diagram below (Figure 4), combining these approaches provides a more comprehensive rehabilitation strategy. The intersection of traditional therapy with neuroscience-guided approaches enables more targeted cognitive and language interventions, while integrating technology-assisted interventions further enhances patient outcomes by leveraging neuroplasticity and AI-driven tools. This multi-modal approach aligns with the growing body of research advocating for personalized rehabilitation strategies that adapt to the individual neuroanatomical and cognitive profiles of PPA patients. By combining these approaches, clinicians can offer more personalized, effective treatments that address the complex cognitive and communication challenges of PPA.

## 4. Discussion

### 4.1. Cognitive Neuroscience Insights in PPA Rehabilitation

A growing number of cognitive rehabilitation studies have explored the restorative potential of primary progressive aphasia (PPA) patients at behavioral level. The relearning mechanisms in PPA are based on the concept of neural communication-driven, and especially synaptogenesis-, gliogenesis-, and neurogenesis-driven, neuroplasticity. The aim of the present study was to analyze the extent to which the neuropsychological rehabilitation of primary progressive aphasia (PPA) has applied cognitive neuroscience insights, beyond just drawing on principles of healthy cognition. PPA is labeled a neurodegenerative condition, and the damage is progressive, which makes it uncertain whether the neurobiological mechanisms of neural relearning observed in the rehabilitation of acquired PPA also apply to primary PPA and to the same extent. Thus far, only one neurobiologically focused study showed a better outcome in the phonological training group, which is consistent with the reduced activation in the left temporal language network reported in functional imaging studies in phonologically impaired versus semantically impaired PPA patients. However, alongside these neuroplastic processes of pruning and declining structural connectivity, functional imaging studies in PPA have revealed the recruitment of a small subset of brain areas in the dominant and non-dominant hemispheres, which could be harnessed to facilitate the behavioral outcome of rehabilitation. Evidence from the neurobiology of memory and restorative learning in acquired aphasia may also be of use in orienting rehabilitation in PPA. Longitudinal, pre-post rehabilitation studies could also provide valuable insights into the potential for neuroplastic adaptation in PPA [5,97,98,99,100,101].

### 4.2. Neuroplasticity Mechanisms

Intuitively, one of the main functions of neuropsychological rehabilitation is to harness the brain’s plasticity. Machine learning meta-analyses supported the idea that plasticity or neuroplasticity incorporates several different mechanisms, such as endophenotypic and adaptive neuroplasticity. In neurodegenerative disorders, definitions of optimal, successful, or more pathological use of plasticity are necessary, which are valuable in explaining non-stable/reduced/gain damages and can guide interventions. Unfortunately, most of the neuropsychological rehabilitations for post-stroke aphasia have not benefited much from cognitive neuroscience advances and have not systematically utilized different types of neural plasticity to guide rehabilitation. In primary progressive aphasia (PPA) patients, their reserve (reserve mechanisms) can help in better conducting compensatory and reserve mechanisms in addition to this pure compensatory mechanism. This also applies to PPA and rehabilitation. For patients diagnosed with PPA, early intensive language training is relatively more common after diagnosis. Previous studies have systematically investigated the neural representations of various language abilities in brain lesions in PPA. Notably, recent neuroimaging studies have also found that aphasia due to stroke can partially restore damaged language-related brain areas through compensation mechanisms. In other words, in PPA neuropsychological rehabilitation, the same language-related areas as stroke aphasia are also expected to have neural reorganization [5,97,99,102].

### 4.3. Theoretical Contributions and Connections

The findings of this systematic review identify the complex interplay between the insights from neuroimaging and neuropsychological rehabilitation perspectives for primary progressive aphasia (PPA). Although it makes a significant contribution to the theoretical landscape, not simply an overview of existing literature, this review synthesizes disparate findings and situates them within established cognitive neuroscience frameworks. One of its major contributions is the introduction of neuroplasticity as a foundational concept underlying language improvement in PPA. Neuronal plasticity, the brain’s ability to adapt and reorganize, underlies the mechanisms by which cognitive and language therapies induce compensatory changes. The present review underscores that the most effective rehabilitation strategies are those that harness neuroplasticity, especially by using targeted interventions tailored to the structural and functional integrity of neural circuits specific to PPA subtypes.

The present research furthers the currently lively theoretical discussion between restorative versus compensatory models of rehabilitation. Under restorative approaches, therapy aims to reactivate dormant but intact neural networks; this is well in line with early-stage interventions in PPA. On the other hand, compensatory strategies, most notably, recruiting alternative pathways, become more applicable as the disease progresses. These dual approaches are further supported by evidence of subtype-specific patterns of atrophy and functional disconnection that determine the neural resources available for recovery. For example, patients with the non-fluent variant (nfvPPA) have been found to take greater advantage of therapeutic approaches targeting the speech production circuitry, whereas the logopenic variant (lvPPA) individuals might respond better to intervention programs based on working memory. This variability underlines the need for precision medicine, in which rehabilitation strategies may be tailored to the unique neural profiles of each subtype of PPA.

It also addresses some of the discrepancies found to date in the literature on the effectiveness of these non-invasive brain stimulation techniques: transcranial magnetic stimulation (TMS) and transcranial direct current stimulation (tDCS). Divergent findings may be systematically contextualized in the larger framework of individual variability of neurodegeneration, regarding baseline atrophy, differences in functional connectivity, and cognitive reserve affecting treatment response. This is in line with new network-based theories of neurodegeneration stipulating that cognitive decline cannot solely be a result of structural atrophy but should also mirror disruptions in dynamic neural network interactions. Such a review, therefore, goes beyond summarizing interventions to critically evaluate their mechanisms and potential limitations based on these theories.

The theoretical contributions of this review are further evidenced in its emphasis on cognitive reserve as a mediating factor in rehabilitation outcomes. According to the cognitive reserve theory, greater neural and cognitive resilience allows the individual to better compensate for brain pathology. This perspective provides a framework for understanding why some patients sustain functional communication despite massive neural loss. The results suggest that interventions to bolster cognitive reserve, such as cognitively stimulating activity or using social support networks, may extend the long-term efficacy of rehabilitation and delay functional decline.

Another important theoretical insight from this review is that PPA provides a model for the study of the dynamic relationship of structural and functional changes in neurodegenerative diseases. Traditional models have generally placed emphasis on the effects of cortical atrophy that drive cognitive decline, though this review highlights the critical role played by functional network disruptions and compensatory mechanisms. Functional connectivity studies have now clearly shown that even when there is severe structural damage, other neural pathways can still be recruited to support residual cognitive functions. This calls for a change in the paradigm of rehabilitation in which not only deficit-based interventions are carried out, but also compensatory mechanisms are enhanced by neurostimulation and focused therapies.

This review also addresses the long-term sustainability of rehabilitation strategies and clinically important questions. While most interventions show initial efficacy, their long-term benefits are less clear, especially in progressive conditions such as PPA. This then raises critical questions about how therapeutic gains can be sustained as the disease advances. Adaptive therapies that evolve together with the changing neurobiological and functional needs of the patient are therefore offered as a solution, informing models that emphasize the dynamic nature of brain-behavior relationships in neurodegenerative diseases.

Finally, this review provides evidence of the importance of incorporating neuroimaging into practice in rehabilitation. Advanced imaging techniques such as MRI, PET, and DTI make it possible for clinicians to better understand the neural substrates underlying language deficits and to develop interventions that target specific brain regions or networks. Indeed, this has brought about a theoretical shift from the traditional one-size-fits-all approach to personalized medicine, where treatment plans are tailored following an individual’s neural and cognitive profile.

In summary, the review synthesizes current evidence but also pushes forward theoretical understanding by connecting the findings to broader frameworks of neuroplasticity, cognitive reserve, and network-based models of neurodegeneration. The work addresses the inconsistencies in the literature, placing emphasis on the necessity of personalized, adaptive interventions and thereby setting a road map for future research and clinical innovation in PPA rehabilitation.

### 4.4. Challenges and Future Directions

It is critical to recognize that these are initial considerations, and the findings reviewed here emphasize that the small sample size and differences between cases make individualized rehabilitation a necessity. We emphasize that the importance of considering the potential for short-term and long-term deficits cannot be overstated, as these can severely complicate an apparent stabilization of language skills [17,24]. For PPA, treatment must also involve addressing the neurodegenerative nature of the clinical condition, and such treatments can be related to multidisciplinary strategies in the future [26,103].

One brain-behavior relation likely to be of importance is the association between individual differences in abilities that support cognitive reserve and the likelihood of a response to rehabilitation. We therefore note that this is an area of research that may open new avenues for the development of treatments, should a larger series of cases show that supporting residual networks can unmask language processing when systems are degraded [104,105,106,107].

Addressing these issues has the potential to extract valuable insights from the success and failure of existing rehabilitation treatments and contributes to a refinement in our understanding of which treatments are most likely to benefit individuals at different stages of the disorder presentation. This is clearly important for PPA, but by examining this evidence, we may also be able to identify treatment candidates in patients who present with related or overlapping symptoms to those described specifically as PPA, thus the impact of these analyses could be far-reaching [108,109,110,111,112,113].

## 5. Conclusions

This comprehensive review underlines the major benefits of the integration of cognitive neuroscience insights—EEG and fMRI, in particular—into neuropsychological rehabilitations of PPA patients, since that could offer important insights not only into the neural mechanisms underlying language deficits but also allow tailored interventions that maximize recovery in PPA patients. These findings recommend that evidence-based guidelines be enacted and uniform training across institutions be utilized to contribute to the patient’s quality of life. In addition, this review emphasizes that more research into finding the long-term efficacy of these neuroimaging-guided therapies is needed, along with refining individualized treatment strategies. This approach, considering the promising bridge between cognitive neuroscience and clinical practice, may constitute a new avenue for managing PPA—a hope toward better therapeutic outcome and sustained language function in affected individuals.

## Figures and Tables

**Table 1:** Research articles of systematic analysis (N = 63).

Authors	Study Objectives	Methodology/Participants	Main Findings
Ash et al., 2019 [34]	- Examine longitudinal changes in language expression during a semi-structured speech sample in patients with primary progressive aphasia (PPA) or behavioral variant frontotemporal dementia (bvFTD) - Relate the longitudinal changes in language expression to longitudinal neuroimaging of cortical thickness	- Longitudinal examination of language expression in a sample of 48 patients with PPA or bvFTD - Assessment of speech fluency and grammar measures, with a focus on decline in grammaticality - Relating longitudinal decline in grammaticality to longitudinal progression of gray matter atrophy in specific brain regions (left frontal operculum/insula and bilateral temporal cortex) - Neuroimaging data were available for a subset of 25 patients	- Patients with different types of primary progressive aphasia (PPA) and behavioral variant frontotemporal dementia (bvFTD) showed declines in speech fluency and grammar, with nonfluent/agrammatic PPA (naPPA) patients declining the most. - The decline in grammatical ability was associated with the progression of gray matter atrophy in the left frontal operculum/insula and bilateral temporal cortex.
Basilakos et al., 2019 [35]	- To evaluate the effect of leukoaraiosis on baseline aphasia severity - To evaluate the effect of leukoaraiosis on long-term changes in aphasia severity	- Retrospective study of 35 participants in the chronic stage (≥6 months) of recovery after a single-event left hemisphere stroke - High-resolution T1- and T2-weighted neuroimaging, with leukoaraiosis severity scored using the Fazekas scale (0–6) - Lesion volume obtained by manually tracing lesions on T2-MRIs and spatially transforming to native T1 space - Language evaluations using the Western Aphasia Battery (WAB) at initial and follow-up assessments, with a mean test-retest interval of 34.8 months	- Leukoaraiosis severity, but not lesion volume, predicted decline in aphasia severity over time. - Lesion volume and time post-stroke were the best predictors of initial aphasia severity. - Leukoaraiosis severity was not a significant predictor of initial aphasia severity.
Berthier et al., 2018 [36]	1. To reappraise mitigated echolalia (ME) in the context of modern neuroscience 2. To report the effects of Constraint-Induced Aphasia Therapy (CIAT) and the drug memantine on detrimental ME in a patient with fluent aphasia 3. To analyze the functional and structural brain correlates of ME in the patient using multimodal neuroimaging	- Language tasks to evaluate mitigated echolalia (ME) in the patient CCR - A placebo-controlled trial with the following phases: - Placebo alone (weeks 0–16) - Placebo + Constraint-Induced Aphasia Therapy (CIAT) (weeks 16–18) - Placebo alone (weeks 18–20) - Washout (weeks 20–24) - Memantine treatment (weeks 24–48) - Instructions to CCR to reduce ME during CIAT - Long-term follow-up with language evaluation and neuroimaging 10 years after treatment	- ME in the patient CCR was associated with deficits in sound-meaning mapping, auditory short-term memory, and impaired inhibition of repetition mechanisms. - CIAT and the drug memantine were effective in reducing the patient’s mitigated echolalia, with the effects lasting for at least 6 months. - The patient’s ME returned to baseline levels 10 years later.
Berthier et al., 2023 [37]	1. To assess the feasibility of a short-term (10-week) intervention trial using Donepezil alone and combined with intensive language action therapy (ILAT) for the treatment of apathy and depression in people with chronic post-stroke aphasia. 2. To evaluate the effectiveness of Donepezil alone and Donepezil combined with ILAT in treating apathy and depression in people with chronic post-stroke aphasia.	- A 10-week intervention trial - Two treatment conditions: Donepezil alone and Donepezil combined with Intensive Language Action Therapy (ILAT) - Sample of 10 people with chronic post-stroke aphasia - Outcome measures: - Western Aphasia Battery - Stroke Aphasia Depression Questionnaire-21 - Neuroimaging assessments (structural MRI and FDG-PET) at baseline and after each treatment endpoint	- The intervention was found to be feasible to implement. - Donepezil alone and combined with ILAT reduced aphasia severity, while apathy and depression only improved with Donepezil-ILAT. - Structural and functional neuroimaging data did not show conclusive results but provide hints for future research.
Caso et al., 2014 [38]	- To identify early cognitive features of sporadic nonfluent/agrammatic primary progressive aphasia (nfvPPA) caused by FTLD subtypes - To identify early neuroimaging features of sporadic nfvPPA caused by FTLD subtypes	- Prospective study design - Collected clinical, neuroimaging, and neuropathological data from 11 patients with sporadic nfvPPA - Divided patients into two subtypes based on underlying FTLD pathology (nfvPPA-tau and nfvPPA-TDP) - Analyzed patterns of cognitive impairment and gray matter (GM) and white matter (WM) atrophy in the whole group and in each pathological subtype separately - Considered longitudinal clinical data	- Apraxia of speech and atrophy in the left posterior frontal lobe were the most common features across the FTLD subtypes studied. - The nfvPPA-tau subtype was characterized by mild to moderate apraxia of speech, mixed dysarthria, agrammatism, and atrophy in the left posterior frontal gray and white matter. - The nfvPPA-TDP subtype was characterized by severe apraxia of speech, spastic dysarthria, mild agrammatism, and atrophy limited to the left posterior frontal gray matter.
Coenen et al., 2019 [39]	-To argue against using generalized data models for DBS surgical targeting due to individual anatomical variability. -To advocate for personalized, patient-specific data in surgical planning, emphasizing that aggregated data may not accurately reflect individual white matter anatomy. -To highlight the limitations of normative data in effectively guiding DBS outcomes and to suggest that tailored approaches could yield better results in complex cases.	-This paper emphasizes the drawbacks of relying on such normative data, arguing for a more individualized approach in DBS decision-making based on unique patient anatomy and brain connectivity rather than generalized datasets.	- The main findings of this paper are that the authors argue against using aggregated normative data for surgical decision-making in deep brain stimulation, as it does not account for individual anatomical variations and potential disease-related changes. They emphasize that surgical targeting and planning should be based on individual patient imaging data, not normative data. The authors also caution against the use of open-source DBS targeting tools like Lead-DBS, as they are not approved for clinical use and can be misinterpreted as providing definitive targeting guidance.
Dazzan et al., 2018 [40]	- To hypothesize that postpartum psychosis (PP) risk may be elevated due to abnormalities in the regulatory T cell (Treg)–CCN protein-(re)myelination axis - To investigate how risk and protective/treatment factors for PP may influence this Treg–CCN-(re)myelination axis - To identify abnormalities in the Treg–CCN-(re)myelination axis that could serve as predictive biomarkers and therapeutic targets for PP	This study examines evidence from both human and animal studies to hypothesize that disruptions in regulatory T cells and immune-mediated myelination processes could elevate the risk of postpartum psychosis in susceptible individuals, particularly those with bipolar disorder or previous postpartum psychosis episodes.	-Immune System Role: Immune system disruptions, specifically in regulatory T cells, are proposed to influence PP risk by impairing brain myelination processes, which are essential for normal brain function during the postpartum period. -CCN Proteins and Remyelination: The study points to CCN proteins as essential mediators in the myelination processes, suggesting that abnormalities here could indicate a novel pathway through which PP develops. -Potential Biomarkers: These immune and myelination pathway abnormalities may serve as predictive biomarkers for PP, offering potential avenues for early detection and intervention.
Di Lorenzo and Muccio, 2023 [41]	- To use tractography and diffusion tensor imaging (DTI) to map the white matter tracts in the brain of a patient with language deficits following a stroke - To investigate the relationship between the patient’s language improvements and the interconnections between cortical and subcortical brain regions - To use multimodal brain imaging (MRI) to evaluate the structural and functional damage caused by the stroke	- Diffusion tensor imaging (DTI) tractography to map white matter tracts in the brain - Structural brain MRI to assess the extent of brain damage - Longitudinal assessment of the patient’s language function over a 6-month period, with DTI tractography performed after the initial 2–3 months of recovery	- Diffusion tensor imaging (DTI) tractography was used to map the white matter tracts in a patient who had a stroke and exhibited language deficits. - The patient developed conduction aphasia due to damage to the arcuate fasciculus, a major white matter tract connecting Broca’s and Wernicke’s areas in the brain. - DTI tractography was used to study the patient’s language recovery process and the interconnections between cortical and subcortical brain regions.
Díez-Cirarda et al., 2018 [42]	- To discuss the current knowledge on the efficacy of cognitive rehabilitation in Parkinson’s disease (PD) - To highlight the next steps that should be taken to fully understand the efficacy of cognitive rehabilitation in PD - To provide an overview of the characteristics of cognitive rehabilitation programs that have been used in PD and the benefits that have been observed - To present the results of a study conducted by the authors on the efficacy of an integrative cognitive rehabilitation program in PD	The study used a group-based structured cognitive rehabilitation program (REHACOP) that targeted multiple cognitive domains, including attention, processing speed, verbal and visual memory, language, executive functions, and theory of mind. The intervention lasted for 3 months, and the researchers evaluated changes in processing speed, visual memory, theory of mind, and functional disability.	- Cognitive rehabilitation programs in Parkinson’s disease have shown efficacy in improving cognitive functions, particularly in the domains of executive functions, working memory, and processing speed. - Cognitive rehabilitation programs in PD can also have a positive impact on quality of life, although the duration of the intervention may be an important factor. - Cognitive rehabilitation programs in PD have been shown to induce brain plasticity and functional brain changes, supporting the idea of a neurobiological basis for the cognitive improvements observed.
Dragoy et al., 2017 [43]	- To report neuropsychological and lesion profiles of 10 new cases of semantic aphasia - To provide support for the relevance of the left TPO area for semantic aphasia using modern neuroimaging techniques - To extend Luria’s neuroanatomical model of semantic aphasia by considering the role of white matter pathways	- Case series of 10 patients with semantic aphasia - Neuropsychological and lesion profile assessments of the patients - Use of modern neuroimaging techniques to examine the involvement of the left TPO area and various white matter tracts (arcuate fasciculus, inferior fronto-occipital fasciculus, inferior longitudinal fasciculus, superior longitudinal fasciculus II and III, and corpus callosum) in the linguistic and non-linguistic deficits of the patients	- The study found that the left temporal-parietal-occipital (TPO) junction is the critical neural underpinning of semantic aphasia, as previously proposed by Luria. - The study also found that white matter pathways, such as the arcuate fasciculus, inferior fronto-occipital fasciculus, inferior longitudinal fasciculus, superior longitudinal fasciculus II and III, and the corpus callosum, are implicated in the linguistic and non-linguistic deficits of patients with semantic aphasia.
Duffau et al., 2021 [44]	- Review new insights into the functional connectome and neural plasticity mechanisms, gained from intraoperative direct electrostimulation mapping and real-time behavioral monitoring in awake patients, combined with perioperative neuropsychological and neuroimaging data. - Explore how these insights can optimize care and rehabilitation for brain-damaged patients, such as in resective oncological or epilepsy neurosurgery and new programs of functional rehabilitation combined with transcranial brain stimulation.	- Intraoperative direct electrostimulation mapping in awake patients - Real-time behavioral monitoring in awake patients - Perioperative neuropsychological assessments - Perioperative neuroimaging data - Longitudinal anatomo-functional correlations to study neural networks and plasticity	- Functional recovery can occur after lesions in presumed “non-compensable” brain areas, challenging the traditional dogma of localizationism. - Intraoperative mapping and monitoring in awake patients have provided new insights into the functional connectome and neural plasticity. - These findings have led to a reappraisal of classical models of cognition, highlighting the dynamic interplay within and between neural circuits, and the importance of subcortical connectivity in limiting neuroplastic potential.
Duncan et al., 2013 [45]	- Discuss advancements in neuroimaging and drug discovery that contribute to understanding pathogenesis - Examine the role of non-pharmacological treatments, including tailored physiotherapy and speech therapy - Emphasize the importance of integrating palliative care within a multidisciplinary approach to treatment.	- The article reviews recent advancements in understanding movement disorders and suggests clinical approaches for geriatricians. It also highlights findings from neuroimaging studies and preclinical research while discussing the integration of palliative care into patient management.	- Movement disorders are no longer considered solely disorders of movement, but are often accompanied by cognitive, neuropsychiatric, and behavioral issues that significantly impact patients and their caregivers. - Significant progress has been made in understanding the underlying pathological mechanisms of movement disorders and in developing new diagnostic tools and drug discovery approaches, but this has not yet led to the development of disease-modifying therapies. - Advances have been made in non-pharmacological interventions like physiotherapy and speech therapy, as well as the increasing recognition of the importance of palliative care in the multidisciplinary management of movement disorders.
Edison, 2023 [46]	- Expand the scope of the journal Brain Connectivity to cover a wider range of topics in clinical neurology, neuroscience, and neuroimaging - Invite submissions focused on: - Neurodegenerative diseases (e.g., Alzheimer’s, Parkinson’s) - Stroke and multiple sclerosis - Mental health - Clinical and translational research - Review articles - Novel neuroimaging techniques and markers - Structural and functional connectivity in brain disorders - Multimodal imaging studies	- This paper discusses the journal’s focus on advancing brain connectivity research across clinical neurology and neuroscience, emphasizing neuroimaging as a critical tool for studying brain structure, function, and disorders.	- Brain Connectivity journal has expanded its scope to cover a wider range of topics in clinical neurology, neuroscience, and neuroimaging. - The journal is interested in publishing articles on various themes related to the expanded scope, including clinical and translational research, review articles, novel neuroimaging techniques, and multimodal imaging studies.
Fickling et al., 2020 [47]	- To investigate whether the combination of physical therapy (PT) and translingual neurostimulation (TLNS) would lead to improvements in cognitive function, in addition to the previously observed improvements in motor function. - To examine event-related potentials (ERPs) using the brain vital signs framework to assess changes in attention and cognitive processing as a result of the PT + TLNS intervention.	- Longitudinal case study design tracking recovery of motor function through multimodal neuroimaging - Use of translingual neurostimulation (TLNS) through the PoNS device to modulate global brain function - Assessment of cognitive function using the brain vital signs framework, which involves a 6-min automated EEG assessment of event-related potentials (ERPs) - Collection of EEG data using a portable 32-channel system and processing to derive the ERP measures	- Physical therapy combined with translingual neurostimulation led to significant improvements in basic attention and cognitive processing compared to physical therapy alone. - The cognitive improvements coincided with a reduction in the participant’s post-traumatic stress disorder symptoms. - The findings suggest the potential importance of non-invasive neuromodulation, like translingual neurostimulation, in cognitive rehabilitation for neurological conditions.
Gangemi et al., 2023 [48]	- To investigate the neurophysiological effects of cognitive rehabilitation (CR) conducted in a virtual environment using the VRRS device - To gain insights into the potential of VR cognitive stimulation as a neurorehabilitation approach for patients with neurological disorders - To contribute to the understanding of the underlying neural mechanisms involved in cognitive improvements from VR-based interventions - To inform the development of innovative and effective interventions to enhance cognitive recovery in individuals with stroke or other neurological events	- Thirty patients with moderate-to-severe ischemic stroke in the chronic phase were enrolled and randomly assigned to either an experimental group (EG) or a control group (CG). - The experimental group received neurocognitive stimulation using virtual reality training (VRT) with the VRRS device, while the control group received the same amount of standard neurorehabilitation using a paper-and-pencil approach. - EEG data were recorded during a 20-min session where the patient was at psychosensory rest, with their eyes closed, to study neuroplasticity changes in theta, alpha, and beta EEG rhythms.	- Virtual reality cognitive training led to significant increases in alpha and beta brain wave activity in the right hemisphere of patients with chronic ischemic stroke, suggesting enhanced neuroplasticity and cognitive functioning. - The control group receiving standard neurorehabilitation did not show any significant changes in alpha and beta brain waves, indicating the effects were specific to the VR intervention. - These findings demonstrate the potential of VR-based cognitive rehabilitation to promote neural plasticity and cognitive recovery in patients with stroke.
Gorno-Tempini et al., 2013 [49]	- To use primary progressive aphasia (PPA) as a model to study the neurobiology of language, including both the cognitive/behavioral and biological aspects - To present a collection of papers on current clinical and research topics related to PPA, ranging from pathological features to novel rehabilitation approaches	- This paper analyzes existing research on PPA, including three main variants—agrammatic, semantic, and logopenic—focusing on how distinct language deficits and associated neurodegenerative patterns can offer insights into language processing and neural network degeneration.	- The paper identifies three main clinical variants of primary Progressive aphasia (PPA), each with distinct speech/language deficits and brain changes, as well as different likelihoods of having frontotemporal or Alzheimer’s pathology. - The paper describes a potential fourth variant of PPA, characterized by milder aphasia and a slower disease progression, within the logopenic variant (lvPPA). - The paper highlights that diffusion tensor imaging (DTI) can be a useful neuroimaging technique for detecting selective white matter changes in the different PPA variants.
Goschke, 2014 [50]	(1) To elucidate the underlying psychological and neurobiological mechanisms of dysfunctions in decision-making and cognitive control in mental disorders. (2) To argue that these dysfunctions represent transdiagnostic mechanisms that may constitute vulnerability factors for a wide range of mental disorders. (3) To identify how different patterns of dysfunction in valuation, cognitive control, and salience processing can lead to distinct symptom profiles across diagnostic categories.	- It provides a selective review of the literature on decision-making, cognitive control, and their relevance for understanding mental disorders. - It does not report any original empirical study, but rather synthesizes existing research on the cognitive, affective, and neural underpinnings of decision-making and cognitive control. - It discusses how dysfunctions in these processes may contribute to the development and maintenance of various mental disorders.	- Dysfunctions in decision-making, volition, and cognitive control, as well as aberrant interactions between the underlying brain systems involved in valuation, performance monitoring, and cognitive control, may represent transdiagnostic mechanisms and vulnerability factors for a wide range of mental disorders. - The specific patterns of cognitive, affective, and motivational dysfunction can vary depending on which processing components are affected (e.g., valuation, cognitive control, salience processing). - These dysfunctions often cut across diagnostic categories, suggesting a need to move beyond symptom-based classifications towards mechanism-based disorder models.
Ho and Nation, 2018 [51]	(1) To investigate the independent and synergistic effects of amyloid beta (Aβ1-42) and phosphorylated tau (Ptau) pathologies on neuropsychological profiles and trajectories (2) To study these effects in cognitively normal older adults	- Used participants from the Alzheimer’s Disease Neuroimaging Initiative (ADNI) who were cognitively normal at baseline - Conducted longitudinal assessment over 36 months, including baseline lumbar puncture and follow-up cognitive exams - Compared different CSF biomarker profiles (Aβ and Ptau) on baseline cognitive performance and trajectories over time - Assessed cognitive domains including memory, attention/executive function, language, and processing speed using various neuropsychological tests - Employed multilevel modeling to analyze the data	- Older adults with both amyloid-beta and phosphorylated tau pathologies showed the worst cognitive performance at baseline, with impairments in memory and executive function. - Older adults with only amyloid-beta pathology also showed cognitive impairments, though not as severe as those with both pathologies. - Older adults with only phosphorylated tau pathology showed slower processing speed compared to those without either pathology.
Holland et al., 2018 [52]	- To explore how the underlying neuropathology (degree of damage to dorsal and ventral language pathways) interacts with the effectiveness of phonological versus semantic therapy for word repetition in individuals with chronic stroke aphasia. - To determine whether an individual would benefit more from restitutive training to restore the function of the damaged neural pathway or compensatory training that takes advantage of the function of the intact neural pathway.	- Recruiting two patients with chronic stroke aphasia and conducting standardized neuropsychological assessments - Developing experimental stimuli that were carefully matched across conditions for each patient - Using a within-subject crossover design with phonological training (audio-visual word presentation) and semantic training (word-picture associations) - Comparing performance on trained items and untrained control items for each training condition	- Both phonological and semantic therapy produced significant gains on trained items for both patients. - Untrained control items showed significant increases only for the phonological condition for patient DM and only for the semantic condition for patient JS, suggesting a double dissociation in generalization effects. - The differential generalization effects were related to the degree of damage to the dorsal and ventral language pathways in each patient, as measured by diffusion tensor imaging.
Iaccarino et al., 2015 [53]	- To conduct 18F-FDG-PET imaging using an optimized SPM procedure at the single-subject and group level in a cohort of clinically diagnosed semantic variant primary progressive aphasia (svPPA) patients - To test the correlation between performance on semantic tasks and specific brain metabolic alterations - To evaluate the relationship between 18F-FDG-PET metabolic patterns and CT atrophy - To test the status of white matter in the uncinate and inferior longitudinal fasciculi and their relationship with 18F-FDG-PET metabolic patterns in a subgroup of patients	- Participants: 10 patients with clinically diagnosed semantic variant of primary progressive aphasia (svPPA) - Neuropsychological assessment: Detailed battery evaluating language, reasoning, memory, and other cognitive domains - 18F-FDG-PET imaging: Acquisition and analysis using SPM12 software at the single-subject and group level - Diffusion Tensor Imaging (DTI): Performed in a subgroup of 3 patients and 20 healthy controls to assess white matter integrity	- The main findings of this study are that semantic variant primary progressive aphasia (svPPA) involves a widespread network of functional derangement, affecting both the temporal lobes and limbic regions, which is associated with both cognitive and behavioral disturbances in patients. - Single-subject PET analysis can help identify the functional abnormalities underlying the cognitive and behavioral symptoms in svPPA patients, which can support the clinical diagnosis. - Convergent evidence from multiple modalities, including clinical, structural, and functional imaging, is important for understanding the neural basis of svPPA and supporting diagnosis in individual patients.
Jobson et al., 2021 [54]	- To discuss the experimental evidence from rodent and non-human primate studies on the role of the medial prefrontal cortex (mPFC) in certain cognitive functions like working memory, decision-making, cognitive flexibility, and attention. - To highlight the key issues regarding the anatomical and functional disparities between rodent and primate studies on the mPFC. - To convey experimental evidence from rodent models of aging and dementia-associated neurological conditions. - To provide an overview of mPFC connectivity in healthy subjects. - To elucidate the functional connectivity differences in aging and dementia-associated disorders in relation to vascular changes measured by resting-state functional MRI.	- Use of animal models, particularly rodents and non-human primates, to study the role of the mPFC in cognitive functions - Review of findings from resting-state functional MRI (rs-fMRI) studies examining functional connectivity changes in the mPFC and associated networks, such as the DMN, in aging and dementia-associated disorders	- The medial prefrontal cortex (mPFC) plays a crucial role in various cognitive functions, and dysfunction of mPFC-related circuits has been implicated in various neurocognitive disorders. - Resting-state functional MRI (rs-fMRI) studies have consistently shown disruptions in mPFC functional connectivity, particularly within the default mode network, across aging and neurocognitive disorders such as Alzheimer’s disease and vascular cognitive impairment. - The paper suggests that these disease-specific patterns of mPFC functional connectivity alterations may serve as potential biomarkers and therapeutic targets for dementia-associated conditions.
Jokel et al., 2016 [55]	- Examine the effects of a naming intervention on naming performance in individuals with semantic variant primary progressive aphasia (svPPA) - Examine the effects of the naming intervention on brain activity in individuals with svPPA	- Four participants with semantic variant primary progressive aphasia (svPPA) - Participants underwent brain scans while performing language tasks - Participants received a 20 h naming therapy intervention based on errorless learning principles - Five healthy control participants underwent brain scans but did not receive any treatment	- Successful re-learning of forgotten vocabulary was accompanied by activation of a larger network in bilateral brain regions in svPPA participants. - The level of activation in the left anterior lobe may be inversely correlated with severity of semantic impairment in svPPA. - Intensive language therapy can lead to behavioral gains and neuroplastic changes even in svPPA patients with advanced anterior temporal lobe atrophy.
Khedr et al., 2014 [56]	- To evaluate the long-term efficacy of dual-hemisphere repetitive transcranial magnetic stimulation (rTMS) on poststroke aphasia - To determine if combining dual-hemisphere rTMS with language training is a feasible treatment for nonfluent aphasia	The methodology of this study involved a randomized controlled trial of dual-hemisphere repetitive transcranial magnetic stimulation (rTMS) combined with speech/language training in 30 patients with subacute poststroke nonfluent aphasia. The real rTMS treatment consisted of 1000 pulses at 1 Hz and 110% rMT over the right unaffected Broca’s area, and 1000 pulses at 20 Hz and 80% rMT over the left affected Broca’s area, for 10 consecutive days, followed by speech/language training. The outcome measures were the language section of the Hemispheric Stroke Scale (HSS), the Stroke Aphasic Depression Questionnaire–Hospital Version (SADQ-H), and the National Institutes of Health Stroke Scale (NIHSS), assessed at baseline, immediately after the 10 sessions, and 1 and 2 months later.	- The real rTMS group showed significantly greater improvements in language function and depression scores compared to the sham rTMS group, and these improvements were sustained for 2 months after the treatment. - The combination of dual-hemisphere rTMS and language training could be a feasible treatment for post-stroke nonfluent aphasia, but further research is needed to confirm these findings.
Leonard et al., 2015 [57]	- To explore the potential influence of “choice” (active engagement of the participant) on the efficacy of the Phonological Components Analysis (PCA) treatment for anomia in individuals with aphasia - To identify the associated neural underpinnings of the PCA treatment, including changes in brain activation patterns	1. Participants: 5 individuals with aphasia were randomly assigned to a Choice or No Choice treatment condition. 2. Treatment: The Phonological Components Analysis (PCA) treatment was used, where participants either chose or were provided the phonological components of target words. 3. Treatment schedule: 30 sessions over 10 weeks, with testing immediately post-treatment and at 4- and 8-week follow-ups. 4. Neuroimaging: 2 participants (1 from each condition) underwent fMRI scanning before and after treatment, completing semantic and phonological judgment tasks.	- The PCA treatment was effective in improving naming performance in all participants, with the effects maintained at 4 and 8 weeks, and some participants also showed generalization to untrained items. - The participant in the Choice condition showed different neural activation changes after treatment compared to the No Choice condition, and these changes were associated with a larger treatment effect and generalization to an untrained task. - This study further supports the efficacy of the PCA treatment, and the neuroimaging data suggests that active engagement in therapy, such as choosing phonological attributes, may engage executive functions that are important for the successful treatment of anomia.
Lockhart et al., 2014 [58]	- Provide an overview of how age-related diseases and genetic factors influence brain aging - Understand how Alzheimer’s disease and cerebrovascular disease contribute to brain aging - Clarify the distinction between “normal” brain aging and the effects of preclinical disease processes	-The paper highlights key themes that cover various neuroimaging techniques and findings related to age-related cognitive decline and associated neurological changes.	- Cognitive aging is a gradual, late-life decline in cognitive performance experienced by most individuals, with age-related deficits in basic cognitive processes and higher-order functions like episodic memory and cognitive control. - Brain structural morphology differs with age, with the most consistent and notable age-related differences in frontal lobar and medial temporal regions, as well as in white matter microstructure. - The study of brain aging must examine the contributions of normal and preclinical disease-related changes, such as Alzheimer’s disease and cerebrovascular disease, to the structure and function of the brain.
Mandelli et al., 2016 [59]	- To test the hypothesis that in non-fluent/agrammatic variant of primary progressive aphasia (nfvPPA), toxic proteins spread transynaptically from an early epicenter to connected regions within the language network, causing the progression of atrophy over time. - To confirm this hypothesis through a longitudinal study, as no previous longitudinal studies have done so.	- Identified an early epicenter of atrophy in 10 early nfvPPA patients using VBM analysis - Derived the functional speech/language connectivity network in healthy controls from this epicenter region and identified the underlying white matter fibers using diffusion tractography - Evaluated the longitudinal changes in gray matter and white matter in 34 nfvPPA patients using VBM longitudinal analysis - Performed correlation analyses between the volume changes in patients and the strength of the healthy functional speech/language network	- Longitudinal gray matter (GM) changes were found in left language-related regions and right inferior frontal gyrus (IFG) and precentral gyrus in non-fluent primary progressive aphasia (nfvPPA) patients. - Longitudinal white matter (WM) changes were found in the underlying regions corresponding to the GM changes. - The pattern of longitudinal atrophy progression in nfvPPA reflects the anatomy of the language functional network, and the spread of degeneration follows specific anatomical and functional network architectures.
Marcotte et al., 2018 [60]	- To identify the effect of treatment intensity (intensive vs. standard) on neural processing associated with word retrieval abilities after PCA treatment - To better understand the brain plasticity mechanisms associated with the intensity of aphasia treatment delivery	- Participants: Two patients with Broca’s aphasia following a stroke in the left middle cerebral artery territory - Treatment: Patients were randomly assigned to either an intensive (10 sessions of 3 h each over 2.5 weeks) or standard (30 sessions of 1 h each over 10 weeks) PCA treatment, with both receiving a total of 30 h of therapy - Neuroimaging: Patients underwent fMRI scans before and after the PCA treatment to examine therapy-induced changes in brain activation during an overt naming task - Stimuli and Assessment: Individualized stimuli were used for the PCA treatment, based on each patient’s performance on a baseline language assessment	- Both short-term intensive and standard, non-intensive PCA treatment improved word retrieval in two chronic aphasia patients, but the improvements were only statistically significant for the patient who received the intensive treatment. - The improvements were long-lasting for both patients, as they maintained the improved naming at 2-month follow-ups. - The neural activation patterns associated with the improved naming differed between the two treatment conditions, with the intensive treatment leading to more decreases in activation, suggesting more efficient processing, while the standard treatment led to more increases in activation, potentially indicating less efficient processing.
Marinescu et al., 2019 [61]	- Develop a new disease progression model called DIVE that can reconstruct long-term brain pathology patterns from short-term longitudinal data - Cluster vertex-wise biomarker measurements to identify areas with similar temporal dynamics and estimate the average trajectory in each cluster - Estimate disease stage and progression speed for each subject, which could be useful for clinical trial stratification and patient management	- DIVE is an image-based disease progression model that operates at the single-vertex resolution on the cortical surface. - It is designed to reconstruct long-term patterns of brain pathology from short-term longitudinal data. - DIVE clusters vertex-wise biomarker measurements (e.g., cortical thickness, amyloid load) that have similar temporal dynamics across patients. - DIVE concurrently estimates an average trajectory of the vertex measurements in each cluster. - DIVE outputs a parcellation of the cortex into areas with common progression patterns, which can serve as a disease signature. - DIVE estimates the disease stage and progression speed for every visit of every subject, which could enhance stratification for clinical trials or management.	- DIVE, a new disease progression modeling method, can identify distinct spatial patterns of pathology progression in different neurodegenerative diseases like typical Alzheimer’s disease and Posterior Cortical Atrophy. - DIVE finds consistent spatial patterns of pathology progression in typical Alzheimer’s disease across different datasets, as well as distinct patterns between different diseases and different biomarker modalities. - The disease stages estimated by DIVE, based solely on imaging data, correlate with cognitive test scores, suggesting the clinical relevance of the method.
Matias-Guiu et al., 2021 [62]	- Evaluate the diagnostic capacity of a connected speech task for diagnosing PPA and its variants - Determine the main components of spontaneous speech - Examine the neural correlates of the spontaneous speech components	- Participants: 118 total, including 31 nfvPPA, 11 svPPA, 45 lvPPA, and 31 healthy controls - Assessments: Cookie Theft picture description task and comprehensive language assessment protocol - Neuroimaging: Patients underwent FDG-PET and MRI - Analyses: Principal component analysis and machine learning to evaluate speech components and diagnostic accuracy, as well as voxel-based analyses to correlate speech components with brain measures	- The connected speech task used in this study was highly accurate in diagnosing PPA and differentiating between its variants, with 91.67% discrimination between patients with PPA and controls and 77.78% discrimination between PPA variants. - Speech rate and lexical features were the most important components of connected speech for diagnosing PPA and its variants. - The lexical component of connected speech was associated with ventrolateral frontal regions, while the fluency component was associated with the medial superior prefrontal cortex, suggesting a subspecialization within the left frontal lobe.
McKenna et al., 2021 [63]	- To evaluate cortical changes in frontotemporal dementia (FTD) patients using both standard cortical thickness analyses and an individualized, z-score-based approach - To determine if standard T1-weighted MRI data from individual patients can be used to generate maps of cortical atrophy, and to assess the potential of this approach to aid in diagnostic classification, clinical decision making, and monitoring longitudinal changes	- Standard cortical thickness analyses - An individualized, z-score-based approach to characterize subject-level disease burden The study found that both of these approaches co detect phenotype-specific patterns of cortical atrophy, and that the quantitative evaluation of individual MRI data may be useful for diagnostic classification, clinical decision making, and assessing longitudinal changes.	- Phenotype-specific patterns of cortical atrophy were detected in FTD patients using both standard cortical thickness analyses and an individualized, z-score-based approach. - The patterns of cortical atrophy observed in each FTD phenotype (bvFTD, ALS-FTD, nfvPPA, svPPA) were consistent with the clinical profiles of those patients. - Individual-level analysis of MRI data can provide valuable insights for diagnostic classification, clinical decision-making, and assessing longitudinal changes in FTD, despite the computational challenges.
Mendes et al., 2024 [64]	- To examine the effects of adding multisite transcranial Direct Current Stimulation (tDCS) to language therapy (LT) for a person with post-stroke non-fluent aphasia.	- Single participant, Mary, with non-fluent aphasia due to a stroke - Participant had received 10 years of personalized language training before the study - Multisite transcranial direct current stimulation (tDCS) was added to the participant’s language training regimen for 15 sessions - Effects were assessed using the Reliable Change Index, which showed improvements in left inferior frontal connectivity, speech production, and comprehension	- The combination of multisite transcranial direct current stimulation (tDCS) and language therapy (LT) improved the participant’s left inferior frontal connectivity and speech production for two months and significantly improved comprehension after one month. - Using multisite tDCS can improve the effectiveness of language therapy for individuals with non-fluent aphasia.
Menéndez-González et al., 2014 [65]	- Explore the use of biomarkers for the early and accurate diagnosis of neurodegenerative disorders - Investigate the role of biomarkers in the differential diagnosis of neurodegenerative diseases - Examine how biomarkers can be used to monitor the progression and follow-up of neurodegenerative disorders	This paper does not describe any specific methodology but rather provides an overview of the different types of biomarker research covered in the special issue, including studies on biochemical/laboratory biomarkers, neuroimaging techniques, multidimensional approaches, and biomarkers for Parkinson’s disease and parkinsonisms.	- Annexin A5 was found to be a good biomarker for both Alzheimer’s disease and Dementia with Lewy Bodies. - CSF biomarkers like Aβ1-42, t-tau, and p-tau can help differentiate Frontotemporal Lobar Degeneration (FTLD) from Alzheimer’s disease (AD), and some FTLD subtypes show unusually low t-tau levels. - APOE ε4 status, but not early-MCI diagnosis, was associated with increased cortical amyloid deposition and lower CSF Aβ levels.
Müller-Dahlhaus et al., 2023 [66]	1. Identifying temporal and spectral signatures of depression in a prefrontal–orbitofrontal–hippocampal network using concurrent TMS-EEG 2. Investigating how these signatures change after rTMS treatment. 3. Exploring the use of TMS-EEG biomarkers to personalize depression treatment.	The key methodological aspects of this study were the use of concurrent TMS-EEG to perturb and probe functional brain networks in individuals with major depressive disorder (MDD) and healthy controls, with a focus on the left dorsolateral prefrontal cortex (DLPFC) and its connections to the orbitofrontal cortex (OFC) and hippocampus (HPC). The study also investigated the effects of two weeks of high-frequency (10 Hz) rTMS targeting the left DLPFC on the DLPFC-OFC-HPC network and depressive symptoms.	- The study found that depression is associated with specific temporal and spectral signatures in a prefrontal-orbitofrontal-hippocampal network, which were normalized after repetitive transcranial magnetic stimulation (rTMS) treatment. - Two weeks of high-frequency rTMS targeting the left dorsolateral prefrontal cortex (DLPFC) renormalized the neural activity and connectivity in the DLPFC-OFC-HPC network, and this was associated with a reduction in depressive symptoms. - The findings suggest that dysfunction in the DLPFC-OFC-HPC network underlies depressive symptoms in major depressive disorder (MDD), and that this network can be targeted and modulated using rTMS to alleviate depressive symptoms.
Musso et al., 2022 [67]	- To develop a language task that could generate discriminative ERPs in patients with aphasia - To determine if the fast word-based BCI task is feasible for patients with aphasia (H1) - To determine if intensive training using the BCI-based feedback can improve language (H2) - To determine if the improvements are language-specific (H3)	- Participants: 10 chronic stroke patients with mild to severe aphasia and 20 healthy controls - Study design: 30 h of BCI-based auditory word detection training over 4 days per week, with language, cognitive, and neuroimaging assessments before, during, and after training - BCI task: Patients performed an auditory word detection task where the BCI provided feedback based on their brain activity to reinforce successful word detection	- The BCI-based language training led to a sustained and generalized recovery of aphasia in patients. - The improvements were specific to language and did not extend to non-linguistic cognitive abilities. - The training-induced recovery was associated with strengthening of the language network and rebalancing of brain networks.
Noort et al., 2019 [68]	1. To provide an overview of studies on the relationship between bilingualism and protection against cognitive decline 2. To investigate whether bilingualism can delay the onset of dementia	The methodology of this systematic review involved searching four major databases for relevant studies, identifying 34 eligible studies (25 original studies and 9 review studies), and categorizing the studies into those investigating bilingualism and cognitive decline in healthy individuals (10 studies) and those investigating bilingualism and onset of dementia (24 studies).	- Mixed results were found regarding the protective effect of bilingualism against cognitive decline, with some studies showing a protective effect and others not. - Several studies found a delay in the onset of dementia of 4–5.5 years in bilingual individuals compared to monolinguals, but this was not a universal finding. - Methodological differences and the complex nature of lifelong bilingualism seem to explain the mixed results, and the authors suggest that large longitudinal studies with objective measurements are needed to draw firm conclusions.
Novakova et al., 2020 [69]	- To assess the effect of rTMS on the activity of task-related brain networks - To identify the neural correlates of the effects of iTBS applied over the left superior parietal lobule (lSPL) using task fMRI	- Twenty healthy young right-handed subjects underwent fMRI before and after rTMS - The fMRI measurement included a Stroop task and resting-state - rTMS was applied using intermittent theta burst stimulation (iTBS) or continuous TBS (cTBS) protocols to the right inferior frontal gyrus (rIFG) or left superior parietal lobule (lSPL) - A crossover design was used with randomized order of stimulation protocols and sites	- A single session of intermittent theta burst stimulation (iTBS) over the left superior parietal lobule (lSPL) led to a significant decrease in activity in the default mode network, particularly in the left anterior cingulate cortex. - The decrease in default mode network activity was specific to the excitatory iTBS protocol over the lSPL, and was not observed with inhibitory continuous TBS (cTBS) or with TBS over the right inferior frontal gyrus.
Osiurak and Massen, 2014 [70]	- Examine the cognitive and neural bases of human tool use - Investigate the nature of the representations and knowledge underlying tool use - Evaluate the competing hypotheses about the role of the inferior parietal lobe in tool use, contrasting the sensorimotor and technical reasoning views	Not mentioned (the paper does not describe a specific methodology, as it is a review article that summarizes the existing literature on the cognitive and neural bases of human tool use)	- The left inferior parietal lobe supports technical reasoning about physical object properties, rather than just sensorimotor knowledge about tool manipulation. - The anterior inferior parietal lobe, particularly the supramarginal gyrus, is involved in understanding mechanical actions, while the posterior parietal cortex is involved in planning the grasping and reaching components of tool use. - When learning a tool use activity, people learn the functional dynamics or mechanical understanding of the task rather than just the specific movements.
Pedersen et al., 2015 [71]	- Review the role of genetics in brain development, structure, and function across the lifespan - Examine the genetic influences on human communication and cognitive abilities - Explore the genetics of dementia and age-related cognitive decline	- Comparative genomics - Developmental perspectives - Linkage studies - Karyotyping - Twin studies - Neuroimaging - Experimental paradigms - Animal models - Genome-wide association studies (GWAS) - Candidate gene studies	- Genetic variation is relevant for understanding individual differences in human traits, from morphological characteristics to behavioral traits. - Heritability of brain structure is generally substantial, and cross-sectional comparisons suggest slight increases from childhood to adulthood, but longitudinal studies are needed to better understand changes over time. - Genetic influences on brain and cognitive aging become stronger as brain resources decrease with age, as described by the resource-modulation hypothesis.
Penner and Sastre-Garriga, 2014 [72]	- To evaluate the efficacy of computer-assisted rehabilitation of attention in multiple sclerosis patients - To compare the effects of a specific computer-assisted attention rehabilitation intervention to a set of non-specific exercises - To assess the impact of the interventions on both cognitive and ecological/perceived outcomes	- A computer-assisted cognitive rehabilitation intervention targeting different attention components, compared to a control group receiving non-specific exercises - Patients were selected based on a clear definition of the cognitive deficit to be targeted by the intervention - The outcome measures used were closely aligned with the targeted cognitive impairment However, the study had some limitations, including lack of a proper placebo arm, incomplete blinding, and lack of a clearly defined primary outcome measure.	- The paper acknowledges that there is some positive evidence for the efficacy of cognitive rehabilitation in multiple sclerosis, including neuroimaging evidence of brain reorganization. - This study found significant improvements in some outcomes for both the intervention and control groups, with larger improvements in the computer-assisted intervention group for certain attention tests.
Powers et al., 2013 [73]	- To relate fractional anisotropy (FA) changes associated with the semantic and logopenic variants of primary progressive aphasia (PPA) to measures of lexical retrieval.	- Recruitment of 24 PPA patients (11 svPPA, 13 lvPPA) and 34 healthy controls - Neuropsychological testing, structural MRI, and DTI data collection for all participants - Use of a tract-specific analysis (TSA) approach to analyze the DTI data and relate white matter changes to lexical retrieval deficits - Assessment of lexical retrieval using category naming fluency (Animals) and confrontation naming (Boston Naming Test)	- Both semantic variant PPA (svPPA) and logopenic variant PPA (lvPPA) showed widespread reductions in white matter fractional anisotropy (FA) compared to healthy controls. - In svPPA, impairments in semantically guided category naming fluency and confrontation naming were related to white matter changes in the left uncinate fasciculus and corpus callosum. - In lvPPA, reduced white matter integrity in the left superior longitudinal fasciculus, left inferior longitudinal fasciculus, and left inferior fronto-occipital fasciculus was related to impairments in confrontation naming and category naming fluency.
Rajagopalan and Pioro, 2019 [74]	- To examine longitudinal changes in brain metabolism (hypo- and hypermetabolism) in a patient with bulbar-onset ALS-FTD - To examine longitudinal changes in brain structure (cortical thickness and cortical surface area) in the same patient - To evaluate how the metabolic changes relate to the structural changes, which can reflect neurodegeneration and neuroinflammation	- Longitudinal 18F-FDG PET and MRI imaging of a single patient with ALS-FTD at baseline and 20.4 months later - Processing of PET and MRI data to obtain measures of cerebral glucose metabolism, cortical thickness, and cortical area, and calculating symmetric percent change between the two time points - Comprehensive neuropsychological testing of the patient at baseline and 20.4 months later to assess cognitive and language function	- Longitudinal 18F-FDG PET revealed both hypo- and hyper-metabolic changes in several brain regions of an ALS-FTD patient with disease progression, which were accompanied by MRI-revealed structural changes. - Structural changes (cortical thinning and changes in cortical area) appeared to precede functional changes (metabolic changes) in some brain regions, while the opposite was true in other regions. - The stage of the underlying neurodegenerative process determines the type and extent of structural changes in hypo- and hyper-metabolic brain regions.
Raji et al., 2014 [75]	- To evaluate the clinical relevance of SPECT in traumatic brain injury (TBI) by reviewing the literature over the past 30 years - To identify whether SPECT can identify TBI, focusing on the anatomical lobar distributions of abnormalities - To compare the identification of abnormalities in TBI on SPECT relative to other modalities like CT and MRI - To assess associations between SPECT abnormalities and neuropsychological/neurological outcomes in longitudinal cohort studies - To further characterize the relationships between SPECT and outcomes in cross-sectional studies	- Conducting a systematic review following PRISMA guidelines - Performing a comprehensive literature search of PubMed and Ovid MEDLINE, supplemented by manual reference searching - Including longitudinal, RCT, and cross-sectional studies, while excluding case reports - Extracting key study details such as sample size, SPECT tracer used, and SPECT lesion localization - Assessing the quality of longitudinal studies using the Newcastle-Ottawa Scale	- SPECT can identify TBI-related abnormalities that are not detected by CT or MRI, particularly in mild TBI cases, and has a high negative predictive value. - SPECT can identify abnormalities in specific brain regions like the frontal and temporal lobes that are not detected by structural imaging, especially in mild TBI. - Longitudinal studies have shown that SPECT can predict clinical outcomes in TBI patients, with abnormal SPECT scans strongly predicting poor outcomes.
Rogalski et al., 2014 [76]	1. Quantify changes in cortical atrophy over 2 years for 3 subtypes of primary progressive aphasia (PPA) 2. Use whole-brain and region-of-interest (ROI) neuroimaging methods to measure the atrophy 3. Quantify disease progression and establish clinical expectations 4. Provide outcome measures for future therapeutic trials in PPA	- Assessing changes in cortical thickness, volume loss, and neuropsychological performance over a 2-year period in 26 patients with 3 different subtypes of primary progressive aphasia (PPA) - Using whole-brain vertex-wise and region-of-interest (ROI) neuroimaging analysis conducted with the FreeSurfer longitudinal pipeline	- All three PPA subtypes (logopenic, agrammatic, and semantic) showed greater atrophy in the left hemisphere compared to the right hemisphere as the disease progressed. - Disease progression was more pronounced within the language network regions of the brain, compared to outside of those regions. - Using a region of interest (ROI) focused on the language network may be a more sensitive way to track disease progression in PPA, compared to whole-brain or ventricular volume measures.
Routier et al., 2018 [77]	- To identify cortical and subcortical brain alterations in the three main variants of primary progressive aphasia (semantic, logopenic, and nonfluent/agrammatic) using multiple neuroimaging modalities (structural MRI, diffusion MRI, and FDG-PET) - To compare the brain alterations across the different PPA variants and across the different imaging modalities	- Participants: 101 subjects (79 PPA, 41 sv-PPA, 26 lv-PPA, 12 nfv-PPA, 22 HC) had T1 MRI and PET, and 77 subjects (59 PPA, 32 sv-PPA, 19 lv-PPA, 6 nfv-PPA, 18 HC) had T1 and diffusion MRI. - T1 MRI analysis: Cortical thickness analysis using FreeSurfer. - Diffusion MRI analysis: Region-of-interest approach using custom pipelines combining tools from FSL, ANTS, and MRtrix, analyzing Fractional Anisotropy (FA) and Mean Diffusivity (MD) within each tract of the JHU WM atlas. - PET analysis: Voxel-based approach using custom pipelines based on SPM, with partial volume correction. - Statistical analysis: General linear model with age and sex as covariates, corrected for multiple comparisons using random field theory for CT and PET and Bonferroni correction for diffusion MRI.	- The semantic variant of primary progressive aphasia (sv-PPA) showed atrophy and hypometabolism in the anterior temporal lobes, particularly on the left side, as well as alterations in white matter tracts connecting these regions. - The logopenic variant (lv-PPA) exhibited more extensive metabolic changes than atrophy, primarily affecting the left temporal–parietal junction and surrounding areas, with corresponding alterations in white matter tracts in this region. - The nonfluent/agrammatic variant (nfv-PPA) showed atrophy and hypometabolism in the left frontal cortex, including Broca’s area, with corresponding white matter tract alterations in the left uncinate fasciculus.
Santos-Santos et al., 2018 [78]	1) To determine the rates of positron emission tomography (PET) amyloid positivity in the main clinical variants of primary progressive aphasia (PPA) 2) To analyze amyloid “discordant” (amyloid positive svPPA and nfvPPA and amyloid negative lvPPA) and mixed cases (PPAm) in search of characteristics that may aid in their identification.	- The methodology of this study involved prospectively recruiting patients with PPA at the UCSF Memory and Aging Center, comprehensively evaluating them clinically and with neuropsychological and language testing, reaching a consensus diagnosis of PPA variant, and performing amyloid PET imaging to assess Alzheimer’s disease biomarker status.	- Primary progressive aphasia variant diagnosis according to the 2011 consensus criteria is highly predictive of Alzheimer’s disease biomarker status, with the logopenic variant being associated with amyloid positivity in over 95% of cases. - Even in cases of semantic and nonfluent/agrammatic PPA with positive amyloid scans, the primary pathology was frontotemporal lobar degeneration with Alzheimer’s disease as a secondary pathology, suggesting that positive amyloid biomarkers do not necessarily rule out a primary FTLD pathology. - The authors conclude that positive amyloid biomarker status does not rule out the possibility of a primary FTLD pathologic process driving the clinical syndrome.
Seeley, 2017 [79]	- Discuss disease onset regions and cell types in more detail - Review neuroimaging data that inform competing models of disease progression - Relate competing concepts of onset and spread to clinicoanatomical convergence and phenotypic diversity - Consider the most important frontiers in selective vulnerability and network imaging	- This paper emphasizes using postmortem studies and in vivo neuroimaging as complementary tools to understand how diseases like Alzheimer’s and Parkinson’s spread within brain networks, guiding future research in network-based disease progression.	- Network-based neuroimaging approaches have provided important insights into the onset and progression of neurodegenerative diseases. - Several fundamental principles of neurodegenerative disease anatomy and pathogenesis have been identified, though some mysteries remain. - The paper discusses how disease patterns are linked to the brain’s network architecture, suggesting a network-based view of neurodegeneration.
Seniów et al., 2013 [80]	The study objectives were to investigate whether repetitive transcranial magnetic stimulation (rTMS) inhibiting the right-hemisphere homologue of Broca’s area improves language restitution when combined with speech/language therapy.	- Randomized controlled trial design with 40 poststroke aphasia patients - Patients were randomized to receive either real or sham rTMS in addition to a 3-week aphasia rehabilitation protocol - Language functions (naming, repetition, and comprehension) were assessed using the Boston Diagnostic Aphasia Examination at baseline, immediately after the 3-week intervention, and 15 weeks after the intervention - The study investigated the effects of inhibitory rTMS applied to the right frontal language homologue, in addition to speech/language therapy, on language recovery in poststroke aphasia patients	- Both the real rTMS and sham rTMS groups showed improvements in language functions after 3 weeks of treatment, with only slight differences in the degree of recovery between the two groups. - For severely aphasic patients, those who received real rTMS showed significantly greater improvement in repetition compared to those who received sham stimulation. - Inhibitory rTMS to the right frontal language area was not effective for all poststroke aphasia patients, but it may benefit some selected patients.
Sharma et al., 2020 [81]	- Identify prognostic factors for neural deterioration in moderate-to-severe traumatic brain injury (TBI) - Inform future intervention research to address modifiable prognostic factors and improve outcomes for moderate-to-severe TBI	- Systematic search of multiple databases (MEDLINE, EMBASE, PsychINFO, CINAHL, SportDiscus, Cochrane) to identify relevant literature - Screening of retrieved studies by two team members - Inclusion criteria focused on studies that examine prognostic factors for neurodegeneration in moderate-to-severe TBI, including longitudinal neuroimaging studies or cross-sectional studies with relevant prognostic factors	Not mentioned (this abstract does not present any main findings or results, as it is a protocol for a planned systematic review rather than a report of an actual study)
Spinelli et al., 2015 [82]	- To longitudinally characterize the clinical and cognitive profile of a case of crossed nonfluent/agrammatic primary progressive aphasia (nfvPPA) that developed into a corticobasal syndrome (CBS) - To investigate the neuroimaging correlates of this case using advanced techniques like 18F-FDG PET, DaT-scan, fMRI, and diffusion tensor MRI	- Longitudinal data collection over 4 years, including: - Clinical and cognitive assessments - 18F-FDG PET neuroimaging - DaT-scan neuroimaging - fMRI during a verb-naming task to assess language lateralization - Diffusion tensor MRI to evaluate white matter damage in the language network	- The patient initially presented with speech impairment and right frontal atrophy. - Over time, the patient developed worsening language deficits, including agrammatism, as well as a left-sided movement disorder. - Neuroimaging showed bilateral language activation and damage to the right superior longitudinal fasciculus.
Suppa et al., 2020 [83]	- To investigate M1 function with TMS during linguistic tasks in FTD patients, including nfv-PPA and bv-FTD - To assess changes in speech-related white matter and grey matter regions using neuroimaging in the same FTD patients	- Transcranial magnetic stimulation (TMS) to examine changes in primary motor cortex (M1) excitability during linguistic tasks in 24 FTD patients (15 with nfv-PPA, 9 with bv-FTD) and 18 healthy subjects - Diffusion tensor imaging (DTI) and voxel-based morphometry (VBM) neuroimaging techniques to assess changes in white matter and grey matter regions involved in language processing in the same FTD patients	- In healthy subjects, transcranial magnetic stimulation (TMS) demonstrated an increase in motor-evoked potential (MEP) amplitudes during specific linguistic tasks, indicating functional connections between speech-related cortical areas and the dominant primary motor cortex (M1). However, this increase in M1 excitability during linguistic tasks was not observed in patients with frontotemporal dementia (FTD), including both the non-fluent variant of primary progressive aphasia (nfv-PPA) and the behavioral variant of FTD (bv-FTD). - This study found decreased fractional anisotropy in the superior and inferior longitudinal and uncinate fasciculi, as well as grey matter volume loss in the left frontal operculum, but not in the parietal operculum or precentral gyrus, in FTD patients. - The white matter and grey matter changes observed were similar between nfv-PPA and bv-FTD, but there was no correlation between the neurophysiological (TMS) and neuroimaging (DTI, VBM) changes in FTD patients.
Szaflarsk et al., 2018 [84]	1. To assess the safety and feasibility of combining intermittent theta burst stimulation (iTBS) and modified constraint-induced aphasia therapy (mCIAT) in patients with post-stroke aphasia 2. To determine if combining iTBS and mCIAT improves language functions after treatment	- Twelve participants underwent fMRI and neuropsychological testing at 3 time points: before the intervention (T1), immediately after the 2-week intervention (T2), and at a 3-month follow-up (T3) - Participants received intermittent theta burst stimulation (iTBS) applied to the language “hot spot” in the left fronto-temporal regions, as determined by fMRI - Participants also received modified constraint-induced aphasia therapy (mCIAT) in group sessions of 3–4 subjects, initiated within 30 min of the first participant receiving iTBS	- The combined intervention of intermittent theta burst stimulation (iTBS) and modified constraint-induced aphasia therapy (mCIAT) was feasible and safe to implement. - The combined intervention led to statistically significant improvements in language function, as measured by the Western Aphasia Battery aphasia quotient (WAB-AQ) and Boston Naming Test (BNT), immediately after the intervention and at the 3-month follow-up. - The observed improvements in language function were associated with changes in brain activity, specifically decreased BOLD signal in the left inferior parietal lobe and right inferior frontal gyrus.
Tao et al., 2021 [85]	1. To examine the neural mechanisms underlying the effects of tDCS when used as an adjunct to behavioral training in neurodegenerative diseases. 2. To specifically examine tDCS-induced neural changes in a language intervention study for primary progressive aphasia (PPA).	- Thirty-two participants with PPA received either tDCS or sham stimulation over the LIFG while undergoing a written naming therapy - Resting-state fMRI data were collected before and after the treatment - The global connectivity of the LIFG-triangularis region was analyzed using the participation coefficient (PC) metric - The PC values were compared between the tDCS and sham groups, as well as with 19 age-matched healthy controls	- Higher global connectivity of the LIFG-tri before treatment was associated with greater dementia severity in PPA patients. - tDCS applied to the LIFG-tri led to a significant decrease in global connectivity compared to sham stimulation. - The decrease in global connectivity was driven by reduced connectivity between the LIFG-tri and regions outside the perisylvian language area, suggesting tDCS enhanced the segregation of the language system and improved processing efficiency.
Tella et al., 2021 [86]	- To identify neural predictors of the efficacy of multimodal rehabilitative interventions in Alzheimer’s disease (AD) continuum patients - To identify the ideal candidates who are most likely to benefit from these rehabilitation treatments	- Inclusion of patients with Alzheimer’s disease (AD) continuum conditions who underwent a multimodal rehabilitation intervention - MRI scans at baseline and neuropsychological evaluations before and after the 8–10 week intervention - Use of FreeSurfer software to extract morphometric measures from the MRI data, including the Medial Temporal Brain (MTB) index, Posterior Brain (PB) index, Frontal Brain (FB) index, and Subcortical Brain (SBCB) index - Logistic regression models to identify predictors of treatment success, including demographic, neural, and neuropsychological variables	- Patients with lower cognitive abilities (MMSE) at baseline but higher brain volume in the posterior brain (PB) regions, especially the right PB, were more likely to show cognitive improvement after the multimodal rehabilitation intervention. - Patients with higher behavioral symptoms (NPI) at baseline, lower brain volume in the frontal brain (FB) regions, and higher brain volume in the PB regions were more likely to show improvement in behavioral symptoms after the rehabilitation intervention. Females were also more likely to benefit from the rehabilitation in terms of behavioral outcomes. - Assessing neural reserve, particularly in the parietal and frontal brain regions, can help identify the patients most likely to benefit from multimodal rehabilitation interventions for Alzheimer’s disease continuum conditions.
Teo et al., 2016 [87]	- Discuss the theoretical framework and evidence for using VR as a therapeutic intervention for neurorehabilitation in various clinical conditions - Provide insights into the efficacy of VR in clinical rehabilitation and its complementary use with neuroimaging and neuromodulation techniques. - Identify areas where more research is needed to understand how different clinical conditions are affected by VR therapies. - Recommend future studies in the form of large, longitudinal, randomized controlled trials to determine the true potential of VR therapies.	- This paper discusses the theoretical frameworks and existing applications of these technologies, highlighting their roles in enhancing neuroplasticity and functional recovery in individuals with motor and cognitive dysfunctions.	- More research is needed to understand how different clinical conditions respond to various VR therapies and how VR can be combined with other technologies like neuroimaging and neuromodulation to enhance its benefits. - Future research should focus on large, long-term studies to better evaluate the effectiveness of VR therapies across different clinical populations. - VR therapies may improve patient engagement and adherence to neurorehabilitation programs by providing objective, quantitative feedback on performance and progress.
Thierry and Rebuschat, 2020 [88]	- To cover a range of topics in the cognitive neuroscience of second and artificial language learning - To examine language learning in diverse populations (children, adults, monolinguals, bilinguals, multilinguals) - To investigate a wide variety of natural and artificial languages, focusing on different language areas (vocabulary, morphology, syntax) - To promote interdisciplinary collaboration and knowledge transfer across research domains in language learning	- Questionnaires and behavioral (psycholinguistic) measures - Event-related potentials (ERPs) - Brain stimulation techniques (e.g., transcranial magnetic stimulation, transcranial direct current stimulation) - Structural and functional neuroimaging (e.g., MRI, PET) - Reaction time experiments - Computational modeling - Eye-tracking - Electrophysiology (e.g., magnetoencephalography)	- The main findings emphasize the complexity of language learning, which involves both internal and external factors, as well as implicit processes that are difficult to measure. - The main findings highlight the need for more sophisticated methods, beyond just behavioral measures, to study language learning and use. - The main findings emphasize the diversity of approaches and methods used in the studies included in this special issue, and how this diversity can lead to cross-disciplinary insights and applications.
Tsapkini et al., 2019 [89]	- Determine if transcranial direct current stimulation (tDCS) over the left inferior frontal gyrus (IFG) can improve semantic fluency in people with primary progressive aphasia, even though fluency was not directly trained. - Use a randomized, double-blind, within-subjects crossover design to evaluate the effects of tDCS on semantic fluency.	- Randomized, double-blind, within-subjects crossover design with two 15-day stimulation periods separated by 2 months - Evaluations performed before, after, 2 weeks post, and 2 months post each stimulation condition - Focused on verbal fluency as an untrained task related to naming - Used leave-one-out cross-validated R-squared to model the additional tDCS effect over sham	- Transcranial direct current stimulation (tDCS) over the left inferior frontal gyrus (IFG) led to significantly greater improvements in semantic fluency immediately after treatment and 2 weeks later, compared to sham stimulation. - The improvements in semantic fluency with tDCS over the left IFG may be related to changes in functional connectivity between the left IFG and other language-related brain regions, such as the left middle temporal gyrus (MTG) and left inferior temporal gyrus (ITG). - The effects of tDCS on semantic fluency are likely due to the left IFG’s role as an important brain region for semantic retrieval, which is a key component of semantic fluency tasks.
Upton et al., 2024 [90]	- To evaluate the efficacy of the iTalkBetter app, a novel digital therapy for speech production in people with chronic aphasia - To identify the brain regions and changes in brain function associated with the use of the iTalkBetter app	- Phase II, item-randomized clinical trial conducted at University College London - Twenty-seven people with aphasia (PWA) used a novel app called “iTalkBetter” that utilizes confrontation naming therapy - Therapy items were individually randomized into “trained” and “untrained” lists, matched on key psycholinguistic variables and baseline performance - PWA practiced with the iTalkBetter app over a 6-week period - Structural and functional MRI data were collected to identify therapy-related changes in brain states - A repeated-measures design was employed	- iTalkBetter significantly improved naming ability by 13% for trained items, with an average increase of 29 words per person, and the benefits persisted for at least 3 months. - Participants’ propositional speech also significantly improved with the use of the iTalkBetter app. - The use of the iTalkBetter app and the amount of practice led to observable changes in the brain structure and function of participants, specifically in the language perception, production, and control networks.
Scuderi and Valenza, 2022 [91]	- Provide an overview of the key facts about Alzheimer’s disease, including the distinction between early-onset and late-onset forms - Discuss the efforts to understand the pathogenesis of AD to develop effective treatments - Highlight the need for further research to understand the complex pathogenesis of AD and identify biomarkers that can detect the disease at early stages	- This paper synthesizes findings from various studies on biomarkers, discussing how specific biomarkers such as amyloid-beta, tau proteins, and neurofilament light chains can serve as diagnostic tools and influence therapeutic approaches. It emphasizes the need for continued development in blood-based and minimally invasive biomarker technologies to improve early diagnosis and treatment monitoring.	- Alzheimer’s disease is a slowly progressive neurodegenerative disease with no available effective treatment, and its prevalence has grown exponentially as life expectancy has increased. - Alzheimer’s disease is characterized by the deposition of amyloid-beta peptides, the formation of neurofibrillary tangles, and neuronal loss, which occur well before the onset of clinical symptoms and progress over decades. - Biomarkers for early diagnosis of Alzheimer’s disease are a major research priority, as they could enable preventive or early treatment, and various approaches, including CSF, blood, genetics, and neuroimaging, are being explored.
Vardy et al., 2017 [92]	- Provide practical suggestions for how to approach and manage cancer-induced cognitive impairment (CICI) in clinical practice - Recommend that oncologists discuss cognitive issues with patients before, during, and after treatment, like how fatigue is discussed - Encourage referral of patients to cognitive studies to better understand the incidence, causes, and treatment of CICI	- Referring patients to participate in cognitive studies to better understand the incidence and causes of CICI - Conducting neuropsychological testing before and after cancer treatment - Referring patients with persistent cognitive symptoms to a neuropsychologist - Evaluating various behavioral interventions for managing CICI, such as education, environmental enrichment, compensatory strategies, and cognitive rehabilitation programs	- Cancer-induced cognitive impairment (CICI) can occur even before cancer treatment and with hormonal treatments, not just with chemotherapy. - While cancer survivors report cognitive symptoms, these do not always correlate with results on formal neuropsychological tests. - The incidence of CICI in survivors of adult solid cancers is unknown, but studies have found cognitive symptoms in up to 70% of patients and objective cognitive impairment in 30–40%, though some studies have not found any impairment.
Vieira et al., 2020 [93]	- To investigate the effects of anodal tDCS over the left ventrolateral prefrontal cortex (lVLPFC) with the cathode over the contralateral supraorbital area (cSOA) on cognitive reappraisal in healthy individuals - To compare the effects of anodal, cathodal, and sham tDCS on cognitive reappraisal in a double-blind, sham-controlled clinical trial	- Participants were recruited through convenience sampling of graduate and post-graduate students over 18 years old, and screened for exclusion criteria. - Participants underwent a single training session for the experimental cognitive task, followed by the experiment 3–5 days later. - Participants were randomly allocated to receive anodal, cathodal, or sham tDCS during the experimental task. - tDCS was applied for 20 min with the anode over the left ventrolateral prefrontal cortex (lVLPFC) and the cathode over the contralateral supraorbital area (cSOA).	- Anodal tDCS over the lVLPFC with the cathode over the cSOA diminished the ability to downregulate negative emotions via reinterpretation in healthy individuals. - This effect was unexpected based on previous studies showing that anodal tDCS over the lVLPFC with the cathode over the right VLPFC facilitated the downregulation of negative emotions. - Participants in the anodal group were unsuccessful in downregulating negative emotions, as there were no significant differences in arousal ratings between the downregulation and maintain conditions.
Vinogradov et al., 2013 [94]	Not mentioned (the abstract does not explicitly state the study objectives)	- Functional magnetic resonance imaging (fMRI) - Diffusion tensor imaging (DTI) - Whole-brain approach to investigate functional and structural connectivity changes in schizophrenia patients after cognitive remediation therapy	- Cognitive remediation therapy has been shown to affect cognition and daily functioning in patients with schizophrenia positively. - However, the underlying neurobiological mechanisms of this treatment are not well understood. - There was previously skepticism about whether cognitive deficits in schizophrenia could be improved through remediation, but some positive findings have been reported. - Concerns have been raised about the methodological rigor and consistency of the evidence on cognitive remediation for schizophrenia.
Woodard, 2017 [95]	- Review new conceptualizations and novel data analytic approaches for longitudinal data - Describe the growth of federally funded longitudinal studies over the last 25 years - Discuss changes in the methods used to analyze longitudinal data	- Reviewing the historical development of longitudinal studies and describing 18 majors federally funded longitudinal studies - Describing the changes in statistical methods used to analyze longitudinal data, transitioning from traditional methods like t-tests and ANOVA to more advanced techniques like latent change scores, linear mixed effects modeling, and latent growth curve models - Discussing changes in the approach to managing missing data in longitudinal studies	- There has been a significant increase in federally funded longitudinal studies in neuropsychology over the past 25 years. - New conceptualizations of longitudinal change have led to the development of novel data analysis approaches. - Researchers should use contemporary statistical methods for analyzing longitudinal neuropsychological data, as they are more powerful and accurate than traditional approaches.
Yang et al., 2019 [96]	- To examine the relationship between longitudinal brain atrophy and semantic deterioration in patients with semantic dementia (SD) - To do this over a one-year period in a cohort of 11 Chinese SD patients	- Longitudinal design following 11 Chinese patients with semantic dementia over 1 year - Used MRI to measure changes in gray matter volume in various brain regions - Examined the relationship between brain atrophy and semantic deterioration in these patients	- The patients’ semantic deterioration was positively associated with gray matter reduction in the bilateral temporal and parietal lobes, including the inferior, middle, and superior temporal gyri, temporal pole, Heschl gyrus, precuneus, and angular gyrus, as well as subcortical regions like the thalamus and putamen. - These posterior temporal and parietal regions were more involved than just the anterior temporal lobes, which are more commonly associated with semantic deficits in semantic dementia. - The authors suggest that longitudinal structural damage in the posterior temporal and parietal regions, in addition to the anterior temporal lobes, plays an important role in the progression of semantic deficits in semantic dementia.

## Abbreviations

PPA	Primary Progressive Aphasia
nfvPPA	Non-fluent/Agrammatic Variant of Primary Progressive Aphasia
svPPA	Semantic Variant of Primary Progressive Aphasia
lvPPA	Logopenic Variant of Primary Progressive Aphasia
FDG-PET	Fluorodeoxyglucose Positron Emission Tomography
MRI	Magnetic Resonance Imaging
fMRI	Functional Magnetic Resonance Imaging
FA	Fractional Anisotropy
MD	Mean Diffusivity
ROI	Region of Interest
TMS	Transcranial Magnetic Stimulation
tDCS	Transcranial Direct Current Stimulation
DTI	Diffusion Tensor Imaging
BCI	Brain-Computer Interface
TDP-43	TAR DNA-binding Protein 43
GRN	Progranulin
CSF	Cerebrospinal Fluid
CAM	Complementary and Alternative Medicine
